# A Cationic Amphipathic Tilapia Piscidin 4 Peptide-Based Antimicrobial Formulation Promotes Eradication of Bacterial Vaginosis-Associated Bacterial Biofilms

**DOI:** 10.3389/fmicb.2022.806654

**Published:** 2022-03-23

**Authors:** Wen-Chun Lin, Yun-Ru Chen, Chi-Mu Chuang, Jyh-Yih Chen

**Affiliations:** ^1^Marine Research Station, Institute of Cellular and Organismic Biology, Academia Sinica, Jiaushi, Taiwan; ^2^Academia Sinica Protein Clinic, Institute of Biological Chemistry, Academia Sinica, Taipei, Taiwan; ^3^College of Nursing, National Taipei University of Nursing and Health Sciences, Taipei, Taiwan; ^4^Department of Obstetrics and Gynecology, Taipei Veterans General Hospital, Taipei, Taiwan; ^5^School of Medicine, National Yang Ming Chiao Tung University, Taipei, Taiwan

**Keywords:** bacterial vaginosis, polymicrobial biofilm, vaginal microbicide formulation, amphipathic antimicrobial peptides, Nile tilapia piscidin 4

## Abstract

Bacterial vaginosis (BV) is prevalent among women of reproductive age and has a high rate of recurrence, which can be largely attributed to ineffective BV biofilm eradication by current first-line antibiotics. In this study, we report that the Nile tilapia piscidin 4 (TP4) exhibits broad-spectrum antimicrobial and antibiofilm activity against BV-associated bacteria, but not beneficial lactobacilli. In addition, BV-associated *Gardnerella vaginalis* remains susceptible to TP4 even after continual exposure to the peptide for up to 22 passages. *Gardnerella vaginalis* and *Streptococcus anginosus* are both biofilm-forming BV-associated bacteria, and we found that combining TP4 peptide and disodium EDTA with the biofilm-disrupting agent, chitosan, can eradicate biofilms formed by single or mixed *G. vaginalis* and *S. anginosus*. In addition, long-term storage of TP4 peptide in chitosan did not diminish its bactericidal activity toward *G. vaginalis*. Preformulation studies were performed using High performance liquid chromatography (HPLC) and Circular Dichroism (CD). The long-term stability of TP4 peptide was assessed under various conditions, such as different temperatures and ionic strengths, and in the presence of H_2_O_2_ and lactic acid. When exposed to sodium dodecyl sulfate (SDS), TP4 maintained its secondary structure at various temperatures, salt and disodium EDTA concentrations. Furthermore, the TP4 microbicide formulation significantly reduced the colonization density of BV-associated bacteria in mice infected with single or mixed bacteria (*G. vaginalis* and *S. anginosus*). The TP4 microbicide formulation showed biocompatibility with beneficial human vaginal lactobacilli and female reproductive tissues in C57BL/6 mice. These results suggest that the TP4 microbicide formulation could be a promising topical microbicide agent for BV treatment.

## Introduction

Bacterial vaginosis (BV) is a common condition in reproductive-aged women, with a worldwide prevalence of over 30%. The cause of BV is microflora imbalance in the vagina, where lactic acid-producing bacteria normally play an essential role of maintaining an acidic environment (pH < 4.5) that inhibits colonization by pathogenic bacteria (O’Hanlon et al., 2013; Borges et al., 2014). One such pathogen is *Gardnerella vaginalis*, which is often increased in BV along with reductions in the proportion of lactic acid-producing bacteria (Deng et al., 2018). Patients typically present with vaginal odor and a thin milky discharge. If treatment is neglected, BV may cause pelvic inflammation, increased susceptibility to preterm birth, or even infertility (Ravel et al., 2021).

Current first-line antibiotic regimens include oral metronidazole, topical intravaginal clindamycin cream, and metronidazole gel (Workowski, 2015). These treatments are associated with a high recurrence rate (over 50% within 6 months to 1 year) for BV that may be due to an inability of antibiotics to effectively eradicate bacterial biofilms (Machado et al., 2016; Hay, 2017). In addition, metronidazole and clindamycin are used to against anaerobic bacteria. However, recent whole-metagenome sequencing studies have identified at least four *G. vaginalis* clades, of which two may be intrinsically resistant to metronidazole (Schuyler et al., 2016). *Gardnerella vaginalis* and BV-associated bacteria often form a complex polymicrobial bio-structure in the BV biofilm (Castro et al., 2019), which is not only composed of anaerobic bacteria. It has been found that some aerobic bacteria, such as *Streptococcus* spp. and *Escherichia coli*, are also involved in BV pathogenesis (Tao et al., 2019). After the patient receives antibiotic treatment, co-existing microbes that are not sensitive to the antibiotics can become dominant species within the human vaginal microbiota. For example, the side effect of antibiotic treatment is vaginal candidiasis (Sobel et al., 2006; Pappas et al., 2016). When patients suffer from vaginitis repeatedly, they are more likely to experience sexually transmitted infections (STI), such as trichomoniasis or human immunodeficiency virus (HIV; Brotman et al., 2010; Cohen et al., 2012), making it difficult to restore a healthy environment. Therefore, new treatment strategies are needed for BV treatment.

Various recent studies have proposed alternative therapeutic approaches to reduce BV recurrence, including extended antimicrobial regimens (Sobel et al., 2006), oral or vaginal administration of probiotic (Bradshaw et al., 2012; Heczko et al., 2015; Marcotte et al., 2019) or amphoteric tenside pessary (WO3191) combined with first-line regimens (Gottschick et al., 2017), and adjunctive intravaginal lactic acid (Plummer et al., 2021) or boric acid treatments (Powell et al., 2019). Although some of these approaches seem promising, there has been limited progress in terms of providing long-term cure after stopping the treatments. Therefore, the active pharmaceutical ingredient (API) for BV treatment should have broad-spectrum antimicrobial activity against relevant vaginal pathogens and not harm the normal beneficial vaginal flora (Mendling et al., 2019). Another consideration is that the broad-spectrum antimicrobial activity API should be used in combination with a biofilm disrupting agent to promote its bactericidal effects.

Antimicrobial peptides (AMPs) are important parts of the innate immune system of many plants and animals, helping to effectively kill invading pathogenic bacteria and regulate host immunity, without harming the host cells (Maróti et al., 2011; Pasupuleti et al., 2012). One group of AMPs has an α-helix configuration with an overall positive charge and amphipathic character (Wang et al., 2016). Because of the positive charge, selective attack of negatively charged pathogenic bacteria is possible. The hydrophobic peptide domains interact with the lipid bilayer to cause membrane disturbances (Toke, 2005; Lázár et al., 2018). In addition, many AMPs can effectively inhibit the formation of biofilms (Yasir et al., 2018). Nile tilapia piscidin 4 (TP4 peptide) is an AMP identified from Nile tilapia (*Oreochromis niloticus*) that exhibits broad-spectrum activity against Gram-positive and Gram-negative bacteria. It is also effective against drug-resistant bacteria (Pan et al., 2015; Hazam and Chen, 2020). Besides its direct actions as a microbicide, TP4 peptide possesses other properties, including regulating host cell immunity, biofilm eradication, and promotion of wound-healing (Huang et al., 2015; Chang et al., 2017; Hazam and Chen, 2020; Liu et al., 2021). These properties may help clear infections, reduce inflammatory responses, and promote restoration of healthy tissues (Liu et al., 2021). However, the TP4 peptide has not been thoroughly tested in the context of BV. In this study, we investigated the therapeutic potential of TP4 peptide in BV. We evaluated the effect of TP4 peptide on survival and biofilm formation of BV-associated bacteria. We also tested combinations of TP4 peptide with a biofilm-disrupting agent on mature biofilms of BV-associated bacteria. Finally, we evaluated the safety and efficacy of the TP4 microbicide formulation *in vivo*, introducing a potential new strategy for BV treatment.

## Materials and Methods

### Peptide Synthesis and Secondary Structure Analysis

Tilapia piscidin 4 peptide (H-FIHHIIGGLFSAGKAIHRLIRRRRR-OH) was synthesized by GL Biochem Ltd (Shanghai, China). The solution structure of TP4 has been reported in a previous study (Protein Data Bank under accession number: 5H2S); peptide structure was visualized using PyMOL (DeLano, 2002).

### Bacterial Strains, Protozoa, and Culture Conditions

Bacterial vaginosis-associated bacteria *G. vaginalis* (ATCC 14018, ATCC 49145), candidiasis pathogen *Candida albicans* (ATCC 14053), trichomoniasis pathogen *Trichomonas vaginalis* (ATCC 30001), and human vagina-derived lactobacilli, *Lactobacillus crispatus* (ATCC 33820), *Lactobacillus gasseri* (ATCC 33323), *Lactobacillus plantarum* (ATCC 14917), and *Lactobacillus jensenii* (ATCC 25258), were purchased from the American Type Culture Collection (ATCC). *Gardnerella vaginalis* M^R^ is a spontaneous metronidazole-resistant mutant of ATCC 14018. The clinical isolates of BV-associated bacteria and healthy human vagina lactobacilli were kindly provided by Dr. Chuang at National Yang Ming Chiao Tung University, Taiwan (all bacterial isolates are listed in Table 1). All BV-associated bacteria were cultured in NYCIII broth (ATCC medium 1685) and grown at 37°C in anaerobic conditions, using AnaeroPack®-Anaero (MGC, Japan). All *Lactobacillus* spp. were cultured in MRS broth (Difco BD) and grown at 37°C in facultatively anaerobic conditions, using AnaeroPack®-MicroAero (MGC, Japan), except *L. gasseri* was grown in aerobic conditions. *Candida albicans* was cultured in YM broth (Difco BD) and grown at 37°C in aerobic conditions.

**Table 1:** Minimal inhibitory concentrations (MICs) and minimum bactericidal concentrations (MBCs) of tilapia piscidin 4 (TP4) and antibiotics tested on clinical isolates of bacterial vaginosis (BV)-associated bacteria and vaginal lactobacilli.

No.	Clinical strain	Metronidazole		Clindamycin		TP4		Biofilm formation ability
		MIC	MBC	MIC	MBC	MIC	MBC	
		μg/ml						
11-1-1	*Streptococcus intermedius*	62.5	250–500	15.63	31.25–62.5	15.63	31.25	X
11-4-1	*Streptococcus anginosus*	125	>500	15.63	62.5–125	7.81	15.63	O
3-1-1	*Streptococcus anginosus*	125	250–500	31.25	125–250	7.81	15.63–31.25	O
3-3-1	*Streptococcus anginosus*	125	250–500	15.63	62.5	7.81	15.63–31.25	O
3-5-8	*Streptococcus anginosus*	>500	>500	31.25	62.5	7.81–15.63	15.63	O
4-3-1	*Streptococcus anginosus*	>500	>500	>500	>500	7.81	7.81–15.63	O
3-2-9	*Streptococcus agalactiae*	>500	>500	500	>500	15.63	15.63–31.25	X
11-5-11	*Streptococcus agalactiae*	>500	>500	125	>500	7.81–15.63	7.81–15.63	X
4-4-1	*Streptococcus agalactiae*	>500	>500	>500	>500	7.81	7.81	O
12-1-1	*Streptococcus pasteurianus*	500	>500	>500	>500	3.91–7.81	15.63–31.25	X
8-1-1	*Escherichia coli*	500	>500	>500	>500	3.91–7.81	15.63	X
8-4-1	*Enterococcus avium*	>500	>500	>500	>500	1.95–3.91	3.91	X
1-3-16	*Actinobaculum schaalii*	15.63	500	>500	>500	1.95–3.91	3.91	X
6-2-4	*Gardnerella vaginalis*	7.81	15.63–31.25	15.63	31.25–62.5	3.91–7.81	7.81–15.63	O
11-5-4	*Gardnerella vaginalis*	62.5	125	500	>500	7.81	7.81–15.63	O
3-2-23	*Gardnerella vaginalis*	3.91	15.63	3.91	3.91–7.81	3.91	7.81	O
4-3-11	*Gardnerella vaginalis*	>500	>500	7.81	15.63	3.91	7.81	O
7-2-4	*Lactobacillus crispatus*	>500	>500	125	250	125	250	X
8-5-12	*Lactobacillus crispatus*	>500	>500	125	250	125	250	X
4-5-8	*Lactobacillus crispatus*	>500	>500	500	>500	62.5	250	X

*X, no biofilm formation ability; O, able to form biofilm*.

### Microbroth Dilution Assay

The antibacterial activities of TP4 and antibiotics were assessed by minimal inhibitory concentration (MIC) and minimum bactericidal concentration (MBC) experiments. The methods for evaluation of *in vitro* antimicrobial activity followed the Clinical and Laboratory Standards Institute (CLSI) guidelines, with minor modifications. Brain-Heart Infusion (BHI) medium (Difco BD) supplemented with 1% glucose (BHIG), RPMI1640 and MRS broth were respectively used for microbroth dilution assays of BV-associated bacteria, *C. albicans* and *Lactobacillus* spp. Serial dilutions were made of the antimicrobial agent with test medium in a round-bottom 96-well microplate (Corning), and the fresh overnight culture of bacterial were added to final inoculum of 5 × 10^5^ colony-forming units (CFU)/ml (1 × 10^3^ CFU/ml in antifungal susceptibility testing, CLSI M27-A3). After a 24-h incubation, the lowest concentration of antimicrobial agent with no visible growth was defined as the MIC. Bacteria were subcultured from broth dilutions in MIC tests on agar plates to determine the lowest concentration of antimicrobial agent that is bactericidal (defined as the MBC).

### Evaluation of Biofilm-Forming Capacity

The biofilm-forming ability of BV-associated bacteria was determined as previously described with minor modifications (Gottschick et al., 2016; Jardak et al., 2017). All BV-associated bacteria were grown in BHI containing 0.3% starch (Sigma) and 1% glucose (sBHI) for biofilm formation. An overnight culture of *G. vaginalis* in growth medium (NYCIII) was subcultured in biofilm medium (sBHI). The subculture was performed several times to allow bacteria to adapt the biofilm medium; the bacteria have different gene expression and metabolism profiles in planktonic and biofilm stages. A fresh overnight culture of bacterial in sBHI was added in 200 μl to final inoculum of 1 × 10^6^ CFU/ml in 96-well flat-bottom microtiter plates (Corning). After a 24-h incubation at 37°C under anaerobic conditions, medium and planktonic cells were removed, and each well was washed twice with saline. After washing, the plates were dried in an oven for 1 h. Biofilms were stained with 200 μl of 0.5% crystal violet solution, then washed twice with 200 μl saline to remove unbound dye. Biofilm-forming ability was determined by visualizing biofilms after crystal violet staining.

### Trichomonacidal Assays

*Trichomonas vaginalis* (ATCC 30001) was maintained in trypticose-yeast-maltose (TYM, ATCC medium 358) media supplemented with 10% (v/v) heat-inactivated bovine serum (Sigma) in 5% CO_2_ at 37°C. For the trichomonacidal assay, 5 × 10^5^ cells were seeded into the wells of a 12-well plate. Then, cells were incubated with the indicated concentrations of TP4 or metronidazole for 24 h. *Trichomonas vaginalis* viability was assessed using the trypan blue exclusion test as previously described (Shaio et al., 1987). A hemocytometer was used to count the number of living cells.

### TP4 Bactericidal Activity in Vaginal Fluid Simulant

**Figure 1:**
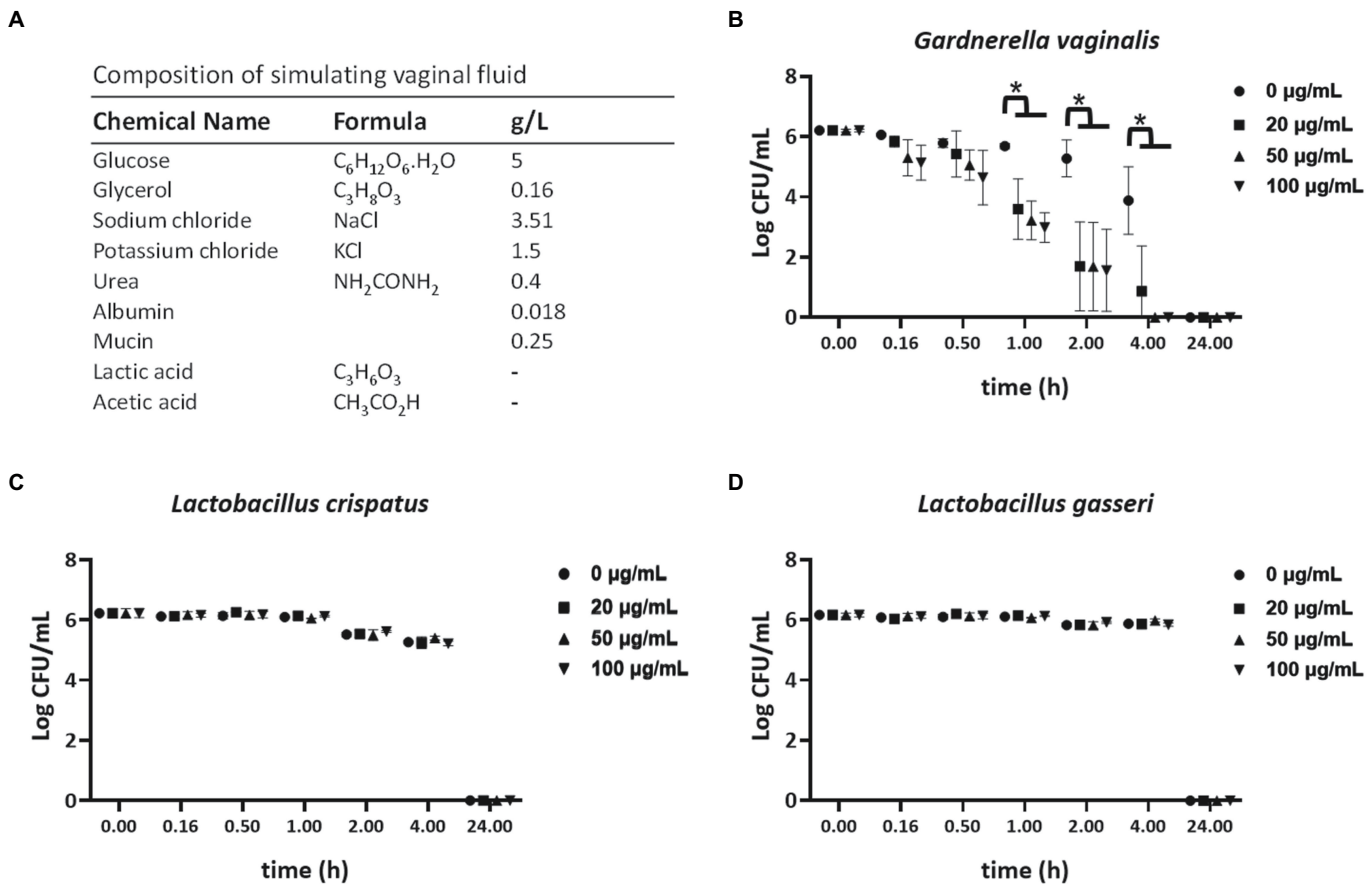
Selective bactericidal activity of TP4 in vaginal fluid simulant (VFS). **(A)** VFS composition. (−): This formula of VFS does not include lactic acid and acetic acid in order to maintain a high survival rate of *Gardnerella vaginalis*. Viability of *G. vaginalis* **(B)**, *Lactobacillus crispatus* **(C)**, and *Lactobacillus gasseri* **(D)** in VFS after treatment with various TP4 concentrations (0, 20, 50, and 100 μg/ml), as determined by colony-forming unit (CFU). All values represent the mean ± SD of three individual experiments. ^*^p < 0.05 compared to CFU/ml from bacteria in VFS TP4-free control, determined by one-way ANOVA.

The vaginal fluid simulant (VFS) composition is shown in Figure 1A, and was prepared as previously described with minor modifications (Tomás and Nader-Macías, 2007). This formula of VFS does not include lactic acid and acetic acid to maintain a high survival rate of *G. vaginalis*. *Gardnerella vaginalis* (ATCC 14018), *L. crispatus* (ATCC 33820), and *L. gasseri* (ATCC 33323) inocula were prepared from fresh overnight cultures adjusted to a final concentration of 1 × 10^6^ CFU/ml in VFS. TP4 was added to a final concentration of 0, 20, 50, and 100 μg/ml (0 μg/ml as control). The mixtures were plated for CFU quantification at different indicated time.

### Time-Kill Analysis

The *G. vaginalis* (ATCC 14018) inoculum was prepared with the fresh overnight culture in BHIG, adjusted to a final concentration of 1 × 10^6^ CFU/ml. TP4 and antibiotics were added to concentrations of 1× MBC (metronidazole: 3.91 μg/ml, clindamycin: 7.81 μg/ml, and TP4 3.91 μg/ml) or 2× MBC (metronidazole: 7.81 μg/ml, clindamycin: 15.63 μg/ml, and TP4 7.81 μg/ml). The antimicrobial agent BHIG was not added for growth control. The number of CFUs was counted from cultures spread onto NYC III agar plates and incubated at 37°C under anaerobic conditions for 0, 0.5, 1, 2, 4, 6, 8, 12, and 24 h.

### Resistance Development Assay

The propensity for TP4 peptide treatment to induce resistance was evaluated by a microbroth dilution assay. *Gardnerella vaginalis* (ATCC 14018) was serially passaged in resistance induction studies. The experiment was performed as previously described with minor modifications (Lee et al., 2018; Elliott et al., 2020). Metronidazole and clindamycin served as antibiotic controls. The MIC test was performed first, and the bacteria were subcultured in half the MIC (1/2 MIC well) with fresh NYC-III medium. After growing bacteria to the stationary phase, the next generation MIC was measured; cells were incubated for 24 h at 37°C, according to the experimental procedure described for the microbroth dilution assay. MICs were determined in triplicate for each of 22 generations.

### Minimal Biofilm Inhibitory Concentration

Evaluation of biofilm formation inhibitory activity was performed as previously described with minor modifications (Algburi et al., 2018). Serial dilutions of the antimicrobial agent were made with sBHI in flat-bottom 96-well microtiter plates, and a fresh overnight culture of bacteria was added to a final inoculum of 1 × 10^6^ CFU/ml. After 24 h of incubation at 37°C under anaerobic conditions, medium and planktonic cells were removed from each well, and the well was washed twice with saline. After washing, the plates were dried in oven for 1 h. Biofilms were stained with 200 μl of 0.5% crystal violet solution, then washed twice with saline to remove unbound dye. Minimal biofilm inhibitory concentrations (MBICs) were determined as the lowest concentration of an antimicrobial agent needed to inhibit the development of biofilms.

### RNA Isolation and Quantitative Real-Time PCR

Fresh overnight cultures of *G. vaginalis* in NYCIII (growth medium) and sBHI (biofilm medium) were used to assess the bacterial cells in planktonic and biofilm stages. Total RNA was extracted using the TRIzol Max Bacterial RNA Isolation Kit (Thermo Scientific) according to the manufacturer’s instructions. Reverse transcription reagent (Toyobo) was used to reverse transcribe total RNA. The expression levels of sialidase, vaginolysin, bacitracin transport ATP-binding protein (BcrA), and multidrug resistance ABC transporter (ABC transporter) gene were analyzed by quantitative real-time PCR (qRT-PCR) using SYBR Green PCR Master Mix (Toyobo) and an ABI StepOnePlus Real-Time PCR System (Applied Biosystems, Foster, CA, United States). The cycling steps were: 5 min at 95°C, followed by 40 cycles of 15 s at 95°C and 60 s at 56°C. The primers used for qRT-PCR are listed in Supplementary Table 1.

### Biofilm Disruption Assay

**Figure 2:**
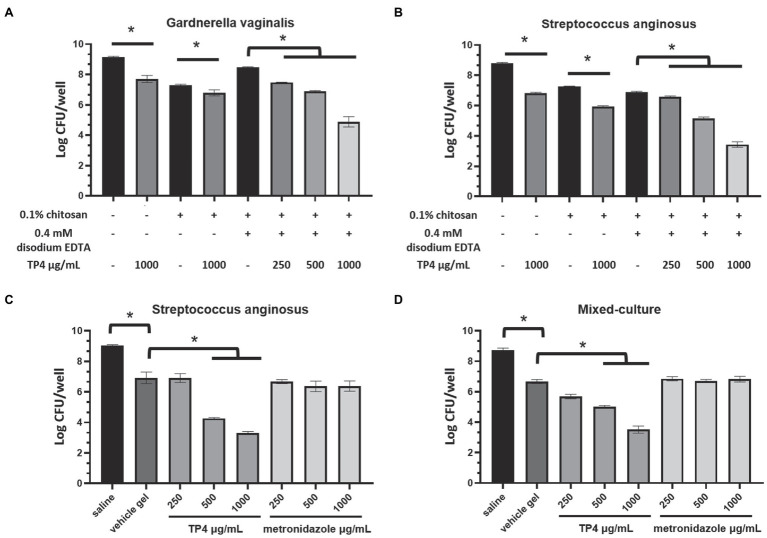
Tilapia piscidin 4 and disodium EDTA formulated in chitosan reduces bacterial viability in BV-associated bacteria biofilms. Mature biofilms formed by BV-associated bacteria *Gardnerella vaginalis* **(A)** or *Streptococcus anginosus* **(B)** were treated with different combinations of TP4, disodium EDTA and chitosan. Mature biofilms formed by **(C)** *S. anginosus* or **(D)** *S. anginosus* combined with *G. vaginalis* (mixed culture) were treated with different concentrations of TP4 or metronidazole in vehicle gel (0.1% chitosan + 0.4 mM disodium EDTA). Biofilm cell viability was measured in terms of CFU counts. All values represent the mean ± SD of three individual experiments (^*^p < 0.05, one-way ANOVA).

The different molecular weights of chitosan (Sigma) used in this study are summarized in Figure 2A. Around 1.2% chitosan stock solution was dissolved in 0.1 M acetic acid in sterile saline. A fresh overnight culture of *G. vaginalis* (ATCC 14018) biofilm was prepared, with planktonic cells removed from each well and washed twice with saline. Subsequently, serially diluted chitosan was added to the mature biofilm. After overnight incubation, the biofilm was washed twice with saline, stained with crystal violet and quantified by OD at 585 nm. Biofilm mass quantification by the crystal violet staining method was performed as previously described with minor modifications (Gottschick et al., 2016).

### Biofilm Cell Viability Assay

A fresh overnight culture of *G. vaginalis* 3-2-23, *S. anginosus* 11-4-1, or *G. vaginalis* 3-2-23 combined with *S. anginosus* 11-4-1 (1:1, mixed-culture biofilm) in sBHI was added to 50 μl of final inoculum of 1 × 10^6^ CFU/ml in a 96-well flat-bottom microtiter plate. After 24 h of incubation at 37°C under anaerobic conditions, medium and planktonic cells were removed, and each well was washed twice with saline. TP4 combined with disodium EDTA (sigma) solution in serial dilutions were added to the mature biofilm. Cells were incubated for 6 h at 37°C in anaerobic conditions, then bacterial viability was analyzed using alamarBlue (Thermo Scientific) according to the manufacturer’s instructions. In the experiment testing TP4 combined with disodium EDTA in chitosan gel against mature biofilm, a fresh overnight culture of *G. vaginalis* 3-2-23, *S. anginosus* 11-4-1, or *G. vaginalis* 3-2-23 combined with *S. anginosus*11-4-1 (1:1, mixed-culture biofilm) in sBHI was added to 400 μl of final inoculum of 1 × 10^6^ CFU/ml in a 24-well flat-bottom microtiter plate. The experimental procedure was performed as described above. Different combinations of TP4, disodium EDTA and chitosan were added to the mature biofilm. After 6 h of incubation at 37°C under anaerobic conditions, bacterial viability was analyzed using the CFU plate count method.

### Preformulation Studies of the TP4 Peptide

High performance liquid chromatography (HPLC) and Circular Dichroism (CD) were used to investigate the stability and secondary structure of TP4 peptide under different environmental conditions. To study the long-term stability of TP4, 500 μg/ml of peptide was dissolved in various aqueous buffer solutions. The influence of temperature of the TP4 peptide was evaluated by storing peptides at −20, 4, 25, 37, and 65°C for 1 month. The effect of salt concentrations on the TP4 peptide was evaluated by storing peptide in 0, 50, 150, 250, 500 mM sodium chloride (NaCl) for 1 month. In the physiological environment, lactobacilli dominate the human vaginal flora, secreting lactic acid and hydrogen peroxide (H_2_O_2_) into the vaginal fluid. The influences of oxidation and acidic environments on TP4 peptide were evaluated. The experimental concentrations were higher than the physiological concentration ranges (O’Hanlon et al., 2011). The effect of acidic environment on TP4 peptide stability was evaluated at 0, 56.5, 113, 565, 1,130 mM lactic acid (physiological concentrations of lactic acid: 55–111 mM; O’Hanlon et al., 2011). The oxidation of TP4 peptide was assessed by exposing TP4 to 8 mM H_2_O_2_ solution (physiological concentrations of H_2_O_2_ < 100 μM) (O’Hanlon et al., 2011). Stability of the TP4 peptide under different environmental conditions was assessed for 1 month; samples were collected on day 0, 1, 2, 3, 7, 14, 21, and 28 for analysis by HPLC (Waters Corporation, Milford, MA) following a previously reported method (Sassi et al., 2011). To study of the secondary structural changes of TP4 peptide and determine its stability in different solutions, TP4 peptide (145 μg/ml) was dissolved in serially diluted membrane-mimicking sodium dodecyl sulfate (SDS) solutions (0, 1, 5, 10, 50, and 100 mM), or 100 mM SDS solution containing different concentrations of NaCl (0, 100, 200, and 400 mM) or different concentrations of disodium EDTA (0, 0.1, 0.4, 1.6, and 6.4 mM). The measure the effects of temperature on the structure of TP4, 145 μg/ml of peptide was pre-incubated at different temperatures (20, 37, 50, and 90°C) in 100 mM SDS solution for 1 h. The far-UV CD spectra of samples were measured using a J-815 CD spectrometer (Jasco, Japan) at 25°C with a wavelength range of 260–190 nm or 260–200 nm. For each spectrum, an average of five scans was collected using a 1 mm path length quartz cell (Hellma Analytics, Germany).

### Experimental Animals

Mouse experiments were approved by the Academia Sinica Institutional Animal Care & Utilization Committee (Protocol number: IACUC 20-12-1568). Six-week-old female C57BL/6 mice were purchased from the BioLASCO Taiwan Co., Ltd. All mice were maintained at a constant temperature and 12:12-h light–dark cycle. Mice were adapted to the environment for 2 weeks before experimentation.

### Mouse Estrus Cycle

The mouse estrus cycle is divided into four main stages, including proestrus, estrus, metestrus, and diestrus, which occur over a period of 4–5 days (Byers et al., 2012). The estrus cycle will affect the physiological characteristics of reproductive tract tissues and local immune response in mice (McLean et al., 2012; Schaefer et al., 2014). At the proestrus to estrus stage, the epithelium is completely keratinized, and superficies are detached gradually in the vaginal cavity (McLean et al., 2012; Schaefer et al., 2014). It is difficult to distinguish epithelial cell detachment caused by physiological or drug toxicity. This stage is therefore not suitable for evaluating drug toxicity. In current studies evaluating the toxicity of topical agents on the reproductive tract, Depo-Provera is administered to maintain the mice in a diestrus-like state, reducing the variability in histopathological evaluations (Ensign et al., 2012; Coulibaly et al., 2019). In the metaestrus to diestrus stage, vaginal epithelial cells become thin, and leukocyte infiltration is present in the vaginal epithelium (McLean et al., 2012; Schaefer et al., 2014). In this stage, the amount of bacteria in the vagina flora is reduced, and the host immune responses prevent bacterial infection and colonization (Jerse et al., 2011; Vrbanac et al., 2018). β-estradiol often used for estrous synchronization in mouse vaginal infection models (Jerse et al., 2011; Gilbert et al., 2013, 2019; Hymes et al., 2013). This treatment dampens the inflammatory response (natural influx of polymorphonuclear leukocytes) and increases susceptibility to bacteria colonization in mouse vagina. Therefore, the mouse vaginal infection model in this study included as step of administering β-estradiol, which kept the mice in an estrus-like state, according to vaginal cytology. This state reduced the variability in bacteria colonization capacity. All mice were confirmed to be at estrus stage according to vaginal cytology before experimentation (Supplementary Figure 5).

### Toxicity Assessment of TP4 Microbicide Formulation in Mice

Evaluation of vaginal toxicity in mice followed a previous study with minor alterations (Ensign et al., 2012; Coulibaly et al., 2019). Eight-week-old female C57BL/6 mice were used to evaluate tissue toxicity after vaginal administration of the TP4 microbicide formulation. To synchronize in the diestrus phase, mice were subcutaneously injected with 2 mg of Depo-Provera (Sigma) in 200 μl of Ringer’s lactate solution, 4 days prior to the drug toxicity test. After synchronization, mice vaginal smears had predominant leukocyte populations, indicating a diestrus-like state. Mice received a single vaginal administration of 20 μl saline (Negative control), 5% Nonoxynol-9 (N-9; Positive control; Sigma), 2% Benzalkonium chloride (BZK; Positive control; Sigma), TP4 at 5 or 10 mg/ml in saline, or TP4 at 5 or 10 mg/ml in gel (vehicle gel contained 0.1% chitosan and 0.4 mM disodium EDTA in saline). “Blank group” means that no drug was administered and there was no irritation of vaginal tissue. Mice were sacrificed at 24 h after drug application for histopathological examination. All animals were sacrificed by exsanguination under anesthesia with pure carbon dioxide. The female reproductive system (vagina, cervix, uterus, and ovary) was collected and preserved in 10% neutral buffered formalin (Sigma). Tissues were trimmed, dehydrated through graded ethanols, cleared in xylene, embedded in paraffin wax, sectioned to approximately 4–5 μm thickness, and stained with hematoxylin and eosin (H&E). Severity of lesions was graded according to the methods described by Shackelford et al. (2002). Degrees of lesions were graded histopathologically from 0 to 5 depending on severity: 0 = normal; 1 = minimal (<1%); 2 = slight (1–25%); 3 = moderate (26–50%); 4 = moderately severe (51–75%); and 5 = severe/high (76–100%).

### Mouse Vaginal Infection Model

In order to test the effects of TP4 microbicide formulation on BV-associated bacteria and normal human vaginal flora *in vivo*, clinical isolates of BV-associated bacteria (*G. vaginalis* 3-2-23 and *S. anginosus* 11-4-1) and *Lactobacillus* spp. (*L. crispatus* ATCC 33820 and *L. gasseri* ATCC 33323) were used to colonize mouse vaginal tissues. The procedures followed a previous study with minor modifications (Gilbert et al., 2013) All bacteria to be inoculated were streptomycin resistant mutants (S^R^) in order to distinguish the inoculated bacteria from endogenous bacteria. Eight-week-old female C57BL/6 mice were subcutaneously injected with 100 μl sesame oil containing 0.5 mg of β-estradiol in the lower abdomen at 1 day and 3 days before the drug administration, in order to synchronize the estrous cycles. Around 2 days before the test drug administration, mice were lavaged and vaginally inoculated with 20 μl of the bacterial suspension once daily for 2 consecutive days. After 2 consecutive days of vaginal inoculation, the test drug was administered twice a day, and the bacterial load was measured by vaginal lavage on the day after administration. Bacterial inoculation doses and the experimental procedure are shown in Supplementary Figure 6. To quantify the bacteria load in the mice vagina after test drug administration, vaginal lavage was performed using a p200 micropipette containing 30 μl sterile saline by gently inserting it into the mouse vagina (about 5 mm deep) and repeated pipetting. The lavage was repeated five times to obtain a total of 150 μl vaginal lavage fluid. The fluid was serially diluted and spread onto NYC III (for counting *G. vaginalis* and *S. anginosus* CFU) or MRS (for counting *L. crispatus* and *L. gasseri* CFU) plates containing 1 mg/ml streptomycin (eliminates endogenous bacteria contamination). The plates were incubated anaerobically at 37°C and colonies were counted after 72 h. All colonies were confirmed by colony PCR. Colony PCR was performed in a Veriti™ 96-Well Thermal Cycler (Applied Biosystems, Foster, CA, United States) with the following cycling parameters: 5 min at 95°C, followed by 40 cycles of 20 s at 95°C, 30 s at 56°C, and 20 s at 72°C. The primers used for colony PCR are listed in Supplementary Table 1.

### Statistical Analysis

GraphPad Prism 8.0 software was used for statistical analyses. Data were collected from at least three independent experiments. Statistical significance was determined by one-way ANOVA with Tukey’s multiple comparison test. Values of *p* < 0.05 were considered significant.

## Results

### Antimicrobial Activity of TP4 Against Pathogens and Normal Vaginal Flora

Current first-line antibiotics for BV are associated with high rates of recurrence and candidiasis. Furthermore, recurrence after antibiotic treatment is associated with increased risk of STIs, such as trichomoniasis (Sobel et al., 2006; Brotman et al., 2010; Cohen et al., 2012). The TP4 peptide has a cationic amphipathic α-helical structure (Supplementary Figure 1) and was reported to exhibit broad-spectrum antimicrobial properties (Hazam and Chen, 2020). To investigate the effects of TP4 on vaginal pathogens, such as *G. vaginalis*, *C. albicans*, and *T. vaginalis*, and healthy human vaginal lactobacilli, the MIC, MBC, and trichomonacidal assay were performed on a panel of vaginal pathogens and lactobacilli. Table 2 and Supplementary Figure 2 show that the control antibiotics exhibited highly potent microbicide activity on vaginal pathogens. The healthy human vaginal lactobacilli showed low susceptibility to antibiotics (MBCs of antibiotics were in the ranges of 1.95–7.81 μg/ml for *G. vaginalis*, <0.48 μg/ml for *C. albicans*, and >62.5 μg/ml for all lactobacilli; Metronidazole <0.98 μg/ml inhibited the growth of *trichomonads*). In contrast, TP4 peptide showed broad-spectrum microbicide activity for all vaginal pathogens, including *G. vaginalis*, *C. albicans*, and *T. vaginalis*, and it was also effective against a metronidazole-resistant strain of *G. vaginalis* (MBC of TP4 peptide against *G. vaginalis*, *G. vaginalis* M^R^ and *C. albicans* were in the range of 3.91–7.81 μg/ml; TP4 peptide 31.25 μg/ml inhibited the growth of *trichomonads*). Notably, all the healthy human vaginal lactobacilli were resistant to TP4 peptide (MBC at >250 μg/ml in all lactobacilli). Next, we assessed the antimicrobial activity of the TP4 peptide on BV-associated bacteria and normal vaginal flora in VFS, which is used to mimic the vaginal environment. Figure 1 shows the survival rate of *G. vaginalis* was decreased by over 90% after a 2-h TP4 treatment (TP4 20, 50, and 100 μg/ml; Figure 1B). In contrast, there were no significant differences in *L. crispatus* (Figure 1C) or *L. gasseri* (Figure 1D) treated with TP4 (TP4 20, 50, and 100 μg/ml) compared to untreated controls. These findings demonstrated the TP4 peptide has broad-spectrum microbicide activity for vaginal pathogens, but it does not negatively affect beneficial vaginal lactobacilli. Importantly, this selective antibacterial activity was also observed in VFS.

**Table 2:** Comparative susceptibility of *Gardnerella vaginalis*, *Candida albicans*, and vaginal lactobacilli to TP4 and antibiotics.

Strain	Metronidazole		Clindamycin		Amphotericin B		TP4		Biofilm formation ability
	MIC	MBC	MIC	MBC	MIC	MBC	MIC	MBC	
	μg/ml								
*Gardnerella vaginalis* (ATCC®14018™)	1.95–3.91	3.91	3.91	7.81			3.91	3.91–7.81	O
*Gardnerella vaginalis* M^R^	>500	>500					7.81	7.81	O
*Gardnerella vaginalis* (ATCC®49145™)	1.95–3.91	7.81	1.95	1.95–3.91			3.91	3.91–7.81	O
*Candida albicans* (ATCC® 14053™)					<0.48	<0.48	7.81	15.63	
*Lactobacillus crispatus* (ATCC®33820™)	>500	>500	62.5	500	>62.5	>62.5	125	250	
*Lactobacillus gasseri* (ATCC®33323™)	>500	>500	>500	>500	>62.5	>62.5	500	500	
*Lactobacillus plantarum* (ATCC® 14917™)	>500	>500	62.5	250			125	250	
*Lactobacillus jensenii* (ATCC® 25258™)	>500	>500	500	>500			250	250	

*M^R^: spontaneous metronidazole-resistant mutant; O: able to form biofilm*.

### TP4 Rapidly Kills *Gardnerella vaginalis* With Low Spontaneous Resistance Frequency

**Figure 3:**
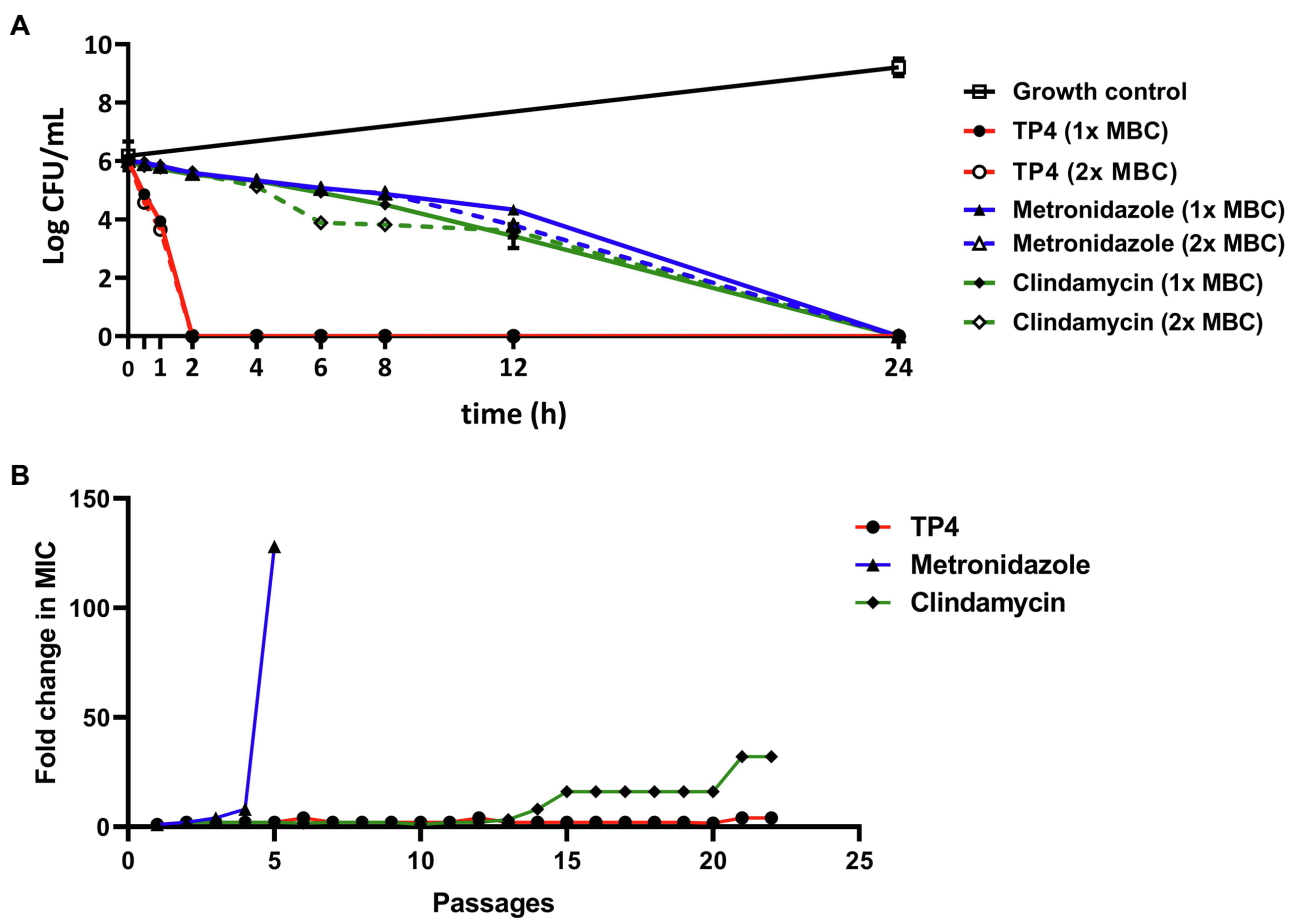
Tilapia piscidin 4 rapidly kills and does not readily induce resistance in pathogenic *Gardnerella vaginalis*. **(A)** Representative time-kill curves for *G. vaginalis* exposed to TP4, metronidazole, or clindamycin at 1× MBC (7.81, 3.91, and 7.81 μg/ml, respectively) and 2× MBC (15.63, 7.81, and 15.63 μg/ml, respectively). The log CFU/ml for all groups were determined at time 0 and then at several time-points up to 24 h. **(B)** Development of resistance in *G. vaginalis* cells exposed to TP4, metronidazole, or clindamycin in an anaerobic environment. The Y-axis shows the fold-change in MIC compared to the first passage. All values represent the mean ± SD of three individual experiments.

The bactericidal mechanism of antimicrobial drugs greatly affects their killing kinetics and resistance development. Therefore, we sought to evaluate the bacterial killing kinetics of TP4 peptides compared with antibiotics. The time-kill kinetics were studied on *G. vaginalis* treated with 1X or 2X the MBC of TP4 peptide or antibiotics. As Figure 3A shows, TP4 peptide killed *G. vaginalis* within 120 min. In contrast, metronidazole and clindamycin exhibited longer bactericidal action times, taking 24 h to kill *G. vaginalis*. We next investigated the propensity for TP4 peptide or antibiotic treatments to induce resistant mutants. Broth dilution was performed to determine the fold-change of MIC after bacterial passage in the presence of microbicides. *Gardnerella vaginalis* was passaged for more than 20 generations during its long-term exposure to TP4 peptide and antibiotics. As shown in Figure 3B, the MIC for metronidazole was increased by more than 100× after four passages (MIC > 500 μg/ml), whereas the MIC for clindamycin was increased 32× after 21 passages (MIC at 125 μg/ml). In contrast, the MIC for TP4 peptide increased only 4× after 22 passages (MIC at 15.63 μg/ml). Based on these data, we conclude that TP4 peptide kill bacteria much faster than first-line antibiotics and it does not readily induce resistance.

### TP4 Prevents BV-Associated Bacterial Biofilm Formation

**Figure 4:**
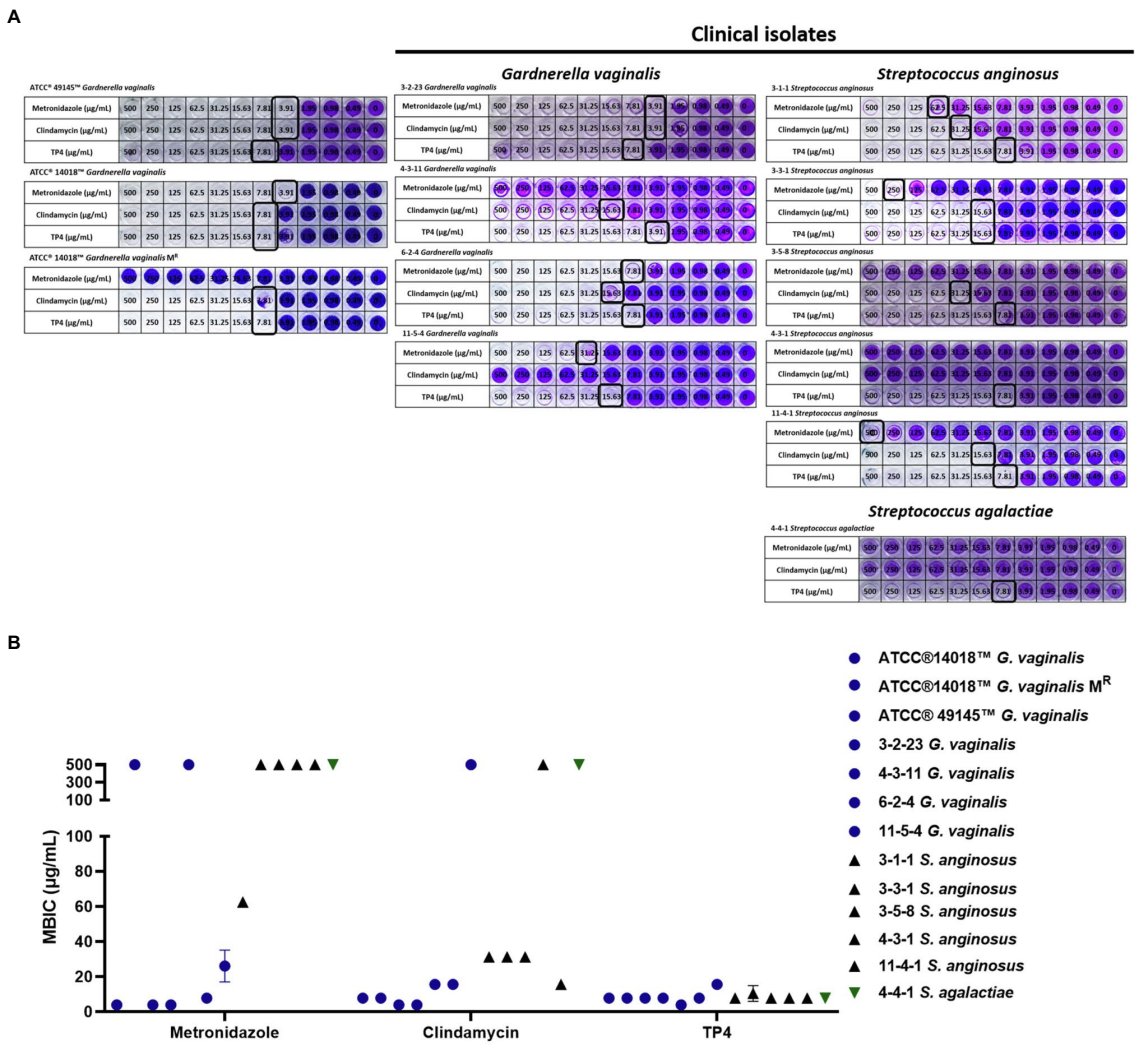
Inhibitory effect of TP4 and antibiotics on BV-associated bacteria biofilm formation. Biofilms formed by BV-associated bacteria were tested with various concentrations of the TP4 and antibiotics. **(A)** The biofilm cells were stained with crystal violet; a representative result from three replicates. Black frame representative Minimal biofilm inhibitory concentration (MBIC). **(B)** Quantification of the MBIC was performed in three individual experiments. The y-axis 500 represents MBIC ≥ 500 μg/ml. All values represent the mean ± SD of three individual experiments.

During our experiments, we noted that *G. vaginalis* has a capacity to form biofilms (Table 2). According to a previous report (Castro et al., 2019), *G. vaginalis* has a greater propensity than other BV-associated species to form biofilms, which function like a scaffold for other BV-associated species to incorporate and colonize the vaginal epithelium. After its formation, other BV-associated bacteria can add biomass to the biofilm, ultimately forming a solid biofilm structure that confers resistance to drugs. Recent studies have found that some aerobic bacteria, such as *Streptococcus* spp. and *E. coli*, are involved in BV pathogenesis (Tao et al., 2019). Therefore, we next evaluated the antibacterial activity of TP4 and antibiotics on clinical isolates of BV-associated bacteria and human vaginal lactobacilli. As shown in Table 1, all *L. crispatus* were resistant to first-line antibiotics and TP4 peptide (MBCs > 250 μg/ml in antibiotics and TP4 peptide to all *L. crispatus*). However, not all the anaerobic bacteria, such as *G. vaginalis*, were sensitive to metronidazole (MICs against *G. vaginalis* ranged from 3.91 to >500 μg/ml). Not surprisingly, the aerobic bacteria, i.e., *Streptococcus* spp., *E. coli* and *Enterococcus avium* were resistant to metronidazole (MICs range from 62.5 to >500 μg/ml). In addition, clindamycin only had a low MIC for some anaerobic bacteria, such as *G. vaginalis*, and aerobic bacteria. Most of the aerobic bacteria and facultative anaerobic *Actinobaculum schaalii* were resistant to clindamycin (MICs against *G. vaginalis* ranged from 3.91 to 500 μg/ml, and MICs against all aerobic bacteria or facultative anaerobic *A. schaalii* ranged from 15.63 to >500 μg/ml). In contrast, all BV-associated bacteria were sensitive to TP4 (MICs against all BV-associated bacteria ranged from 3.91 to 15.63 μg/ml). We observed that among BV-associated bacteria, *S. anginosus*, *S. agalactiae*, and *G. vaginalis* have biofilm-forming capacities (Table 1). Thus, we next used crystal violet staining to evaluate whether TP4 peptide and antibiotics can prevent BV-associated bacteria biofilm formation. As shown in Figure 4, metronidazole and clindamycin only partially prevented BV-associated bacteria biofilm formation. In contrast, TP4 peptide prevented all the BV-associated bacteria from forming biofilms. MBICs for metronidazole ranged from 3.91 to >500 μg/ml; MBICs for clindamycin ranged from 3.91 to >500 μg/ml, and MBICs for TP4 peptide ranged from 3.91 to 15.63 μg/ml. These results showed that first-line antibiotics cannot prevent all BV-associated bacteria from forming biofilms. However, TP4 can prevent all biofilm formation by all BV-associated bacteria. Most importantly, the data also revealed that *L. crispatus* clinical isolates are tolerant to TP4 peptide.

### Biofilms Increase Antibiotic Resistance Development and Associated Gene Expression in *Gardnerella vaginalis* Isolates

In general, biofilms are much more tolerant to antimicrobial agents than planktonic cells, which may a cause for failure of antibiotic treatments and disease recurrence (Hall and Mah, 2017). Biofilm-forming capacity is regarded as a virulence factor for BV pathogenesis, so we compared the biofilm-forming capacities of different *G. vaginalis* isolates. There were no significant differences in biofilm formation between different *G. vaginalis* isolates cultured in the same culture conditions (sBHI, anaerobic culture for 24 h; Supplementary Figure 3A). We then wanted to gather information regarding the regulation of virulence gene expression in *G. vaginalis* biofilms. We observed that in most *G. vaginalis* isolates, expression of the sialidase gene and antibiotic resistance-associated genes, such as multidrug resistance ABC transporter and BcrA were significantly increased in biofilms over planktonic cultures. Meanwhile, the cytotoxicity associated gene, vaginolysin, was significantly decreased in the biofilms compared to planktonic cells (Supplementary Figure 3B). These results suggest that biofilm formation may increase resistance to the intracellular actions of antibiotics.

### Chitosan Disrupts Biofilms and can be Used as an Excipient for TP4

**Figure 5:**
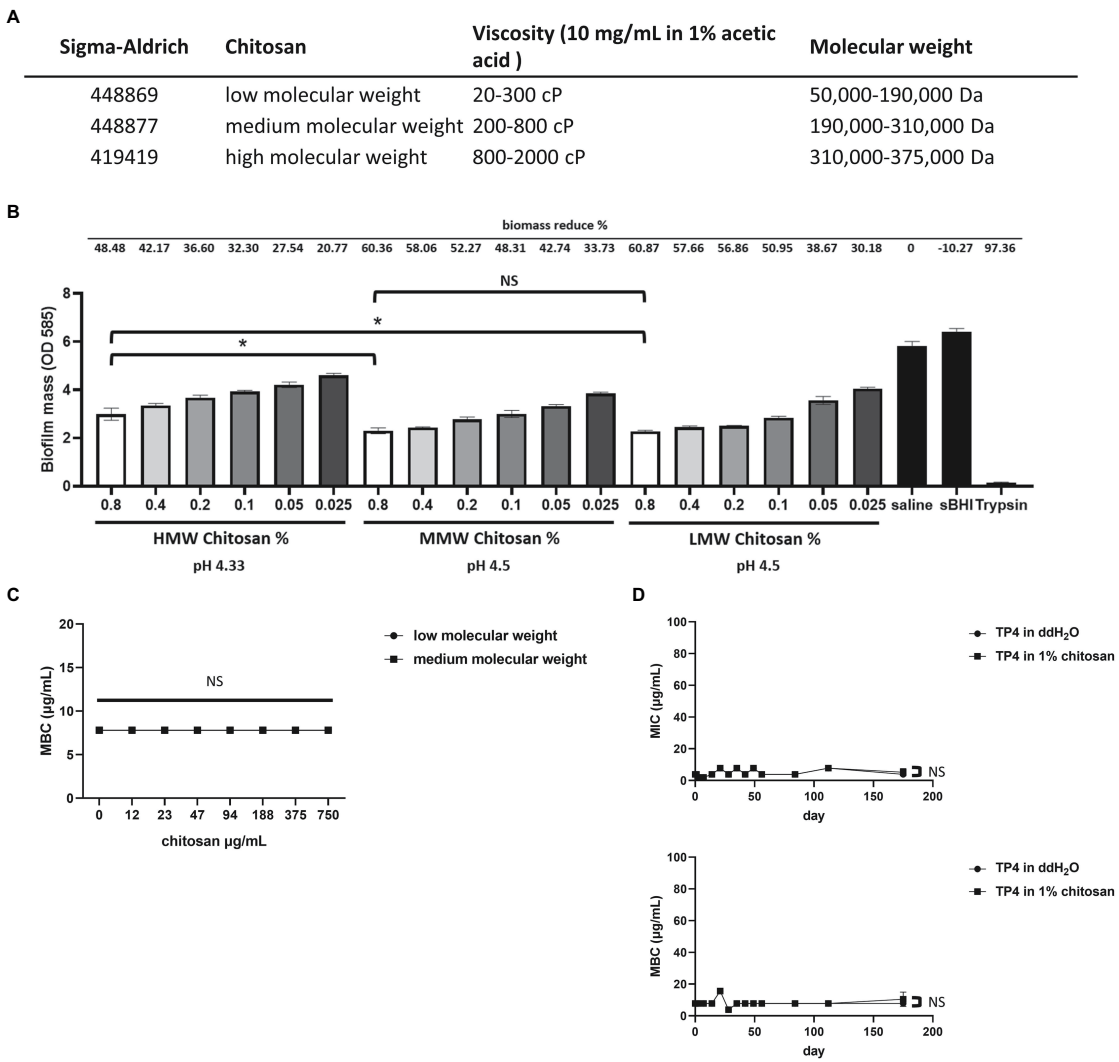
Chitosan can disrupt *G. vaginalis* biofilm and does not affect TP4 bactericidal activity. **(A)** Different molecular weights of chitosan were used. **(B)** Testing the biofilm disruption activity of different molecular weights of chitosan. Different molecular weights of chitosan were added to *Gardnerella vaginalis* biofilms at different concentrations. Saline served as a vehicle control; sBHI served as a negative control; trypsin served as a positive control. Biofilm mass was determined by crystal violet staining. Data show the quantified biofilm mass and average biomass reduction (%) from three individual experiments. **(C)** TP4 bactericidal activity in Brain-Heart Infusion glucose (BHIG) with different concentrations of low molecular weight (LMW) and medium molecular weight (MMW) chitosan. **(D)** Long-term stability of TP4 stored in chitosan gel was analyzed over time. TP4 was dissolved in chitosan gel or ddH_2_O. MICs and MBCs were determined at different time-points. All values represent the mean ± SD of three individual experiments (^*^p < 0.05, one-way ANOVA). NS, no significant differences.

Chitosan is a positively charged linear polysaccharide that is widely used in the biomedical and biotechnological fields due to its non-toxic, stable, biodegradable characteristics (Bhattarai et al., 2010). Recent studies showed that chitosan disrupts *Staphylococcus* biofilms (Felipe et al., 2019) and exhibits antifungal activity (Lo et al., 2020). To investigate whether chitosan can also disrupt *G. vaginalis* biofilm activity, we cultured biofilms with different molecular weights of chitosan (Figure 5A). Figure 5B shows that various molecular weights of chitosan disrupt *G. vaginalis* biofilm formation in a dose-dependent manner. Notably, low molecular weight (LMW) and medium molecular weight (MMW) chitosan more significantly reduced *G. vaginalis* biomass than high molecular weight (HMW) chitosan (significant difference between HMW and MMW/LMW chitosan at 0.8%; no significant differences between LMW and MMW). Next, we investigated whether the LMW and MMW chitosan would affect TP4 peptide antimicrobial activity. TP4 peptide was prepared in chitosan gel and used to determine *G. vaginalis* MBC values. Figure 5C shows that LMW and MMW chitosan do not affect TP4 peptide antimicrobial activity (MBCs of 7.81 μg/ml at different concentrations of chitosan are the same as TP4 peptide dissolved in water). We then tested whether TP4 peptide stored long-term in MMW chitosan gel was stable and retained antimicrobial activity. TP4 peptide was stored in MMW chitosan gel for half a year, during which the MIC and MBC were determined at different time points. As Figure 5D shows, the MICs and MBCs were not significantly different between TP4 peptide in MMW chitosan gel or dissolved in water. There was no apparent change in MICs or MBCs with prolonged storage times (MICs remained in the range of 1.95–7.81 μg/ml, and MBCs remained in the range of 3.91–15.63 μg/ml). Taken together, these results showed that TP4 peptide can be stored in MMW chitosan gel for months without affecting its antimicrobial activity. Furthermore, the biofilm-disrupting properties of chitosan may allow it to serve as an excipient for TP4 peptide.

### TP4 Peptide Combined With Chelating Agent Disodium EDTA and Chitosan Promotes Eradication of Biofilms Formed by BV-Associated Bacteria

The inability to eradicate polymicrobial BV biofilms may be largely responsible for the high disease recurrence rates associated with current first-line regimens antibiotics (Machado et al., 2016). We found that BV-associated bacteria, *G. vaginalis* and *S. anginosus*, exhibit strong biofilm-forming capacities (Table 1). To investigate the impact of the TP4 peptide on BV-related biofilms. *Gardnerella vaginalis*, *S. anginosus*, and mixed-culture biofilm (*G. vaginalis* combined with *S. anginosus*) were subjected to testing. EDTA is a Food and Drug Administration (FDA) approved preservative for pharmaceutical products, as it can chelate divalent ions. EDTA can destabilize the biofilm matrix and enhance susceptibility to antibacterial agents (Cavaliere et al., 2014; Field et al., 2017). Checkerboard assays showed (Supplementary Figure 4) that low concentration disodium EDTA (below 0.8 mM) enhanced TP4 bactericidal activity in *G. vaginalis*, *S. anginosus*, and mixed-culture biofilms (disodium EDTA >6 mM had an antagonistic effect on TP4 microbicide activity; data not shown). Next, we evaluated the bactericidal activity of TP4 peptide combined with disodium EDTA and chitosan in mature biofilms. Figure 2 shows that TP4 peptide combined with disodium EDTA and chitosan significantly decreased the number of CFU from *G. vaginalis* (Figure 2A), *S. anginosus* (Figures 2B,C), or mixed-culture biofilms (Figure 2D) in a dose-dependent manner (TP4 0, 250, 500, and 1,000 μg/ml). In contrast, metronidazole is inactivated when used on *S. anginosus* (Figure 2C) or mixed-culture biofilms (Figure 2D; no significant difference between metronidazole and vehicle). These results suggest that a TP4 microbicide formulation consisting of TP4 peptide, disodium EDTA and chitosan can promote the eradication of BV-associated bacteria biofilms.

### Preformulation Studies on TP4 Peptide

**Figure 6:**
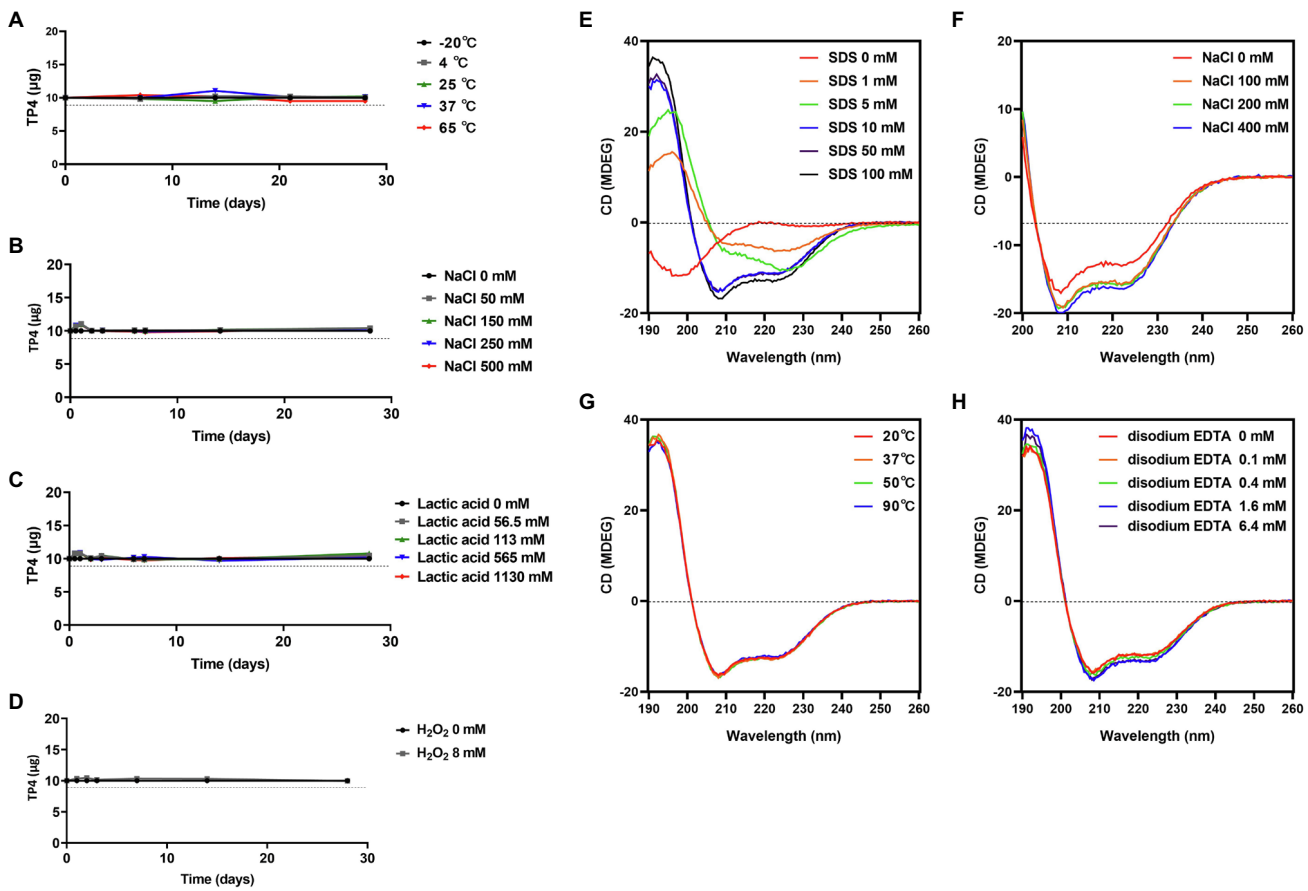
Preformulation studies on TP4 peptide. High performance liquid chromatography (HPLC) of 500 μg/ml TP4 peptide dissolved in various aqueous buffer solutions or conditions and kept for various times. **(A)** TP4 was stored at −20, 4, 25, 37, and 65°C to evaluate the effects of temperature. **(B)** TP4 was incubated in different NaCl concentrations (0, 50, 150, 250, and 500 mM) to evaluate effects of ionic strength. **(C)** TP4 was stored in different lactic acid concentrations (0, 56.5, 113, 565, and 1,130 mM) to evaluate the effects of acidic physiological conditions. **(D)** TP4 oxidation was evaluated in 8 mM H_2_O_2_ solution. The dotted line represents 90% stability. Data are representative of three independent experiments and values are expressed in mean ± SD. Circular Dichroism (CD) spectra of 145 μg/ml TP4 peptide dissolved in the indicated concentrations of sodium dodecyl sulfate (SDS; **E**), in 100 mM SDS plus the indicated concentrations of NaCl **(F)**, in 100 mM SDS at 20, 37, 50, and 90°C for 1 h **(G)**, or in 100 mM SDS plus the indicated concentrations of disodium EDTA **(H)**.

Tilapia piscidin 4 peptide has broad-spectrum activity against BV-associated bacteria and is a promising agent in the treatment of BV. However, peptide drugs are susceptible to environmental influences, such as excipients or vaginal fluid that may cause conformational changes, oxidation or hydrolysis, affecting the antimicrobial activity. Long-term stability studies were performed on TP4 peptide exposed to different temperatures or different concentrations of salt, lactic acid, and oxidation conditions using HPLC. No detectable differences were observed for TP4 peptide stored at −20, 4, 25, 37, and 65°C for 28 days (Figure 6A). In addition, a healthy vagina is dominated by lactobacilli that produce H_2_O_2_ (physiological concentrations < 100 μM) and lactic acid (physiological concentrations 55–111 mM) to eliminate other pathogens (O’Hanlon et al., 2011). Long-term stability studies showed that TP4 peptide samples exposed to a range of H_2_O_2_ (0, 8 mM), lactic acid (0, 56.5, 113, 565, and 1,130 mM) and NaCl (0, 50, 150, 250, and 500 mM) concentrations were unaffected after 28 days (Figures 6B–D). This result suggests that TP4 peptide is stable in the presence of lactic acid and H_2_O_2_ as well as at a wide range of temperatures and ionic strengths. This is an important finding, as the drug will be applied in a vaginal environment (typical pH 3.5–4.5, H_2_O_2_ < 100 μM). In order to investigate potential secondary structure and conformational changes in the TP4 peptide, CD experiments were conducted under several conditions. As illustrated in Figure 6E, TP4 peptide dissolved in H_2_O exhibited negative peaks at around 198 nm in the CD spectra (SDS 0 mM), suggesting the presence of a random coil structure. With increasing SDS (membrane-mimicking condition) concentrations, a strong positive peak appeared at 191 nm, accompanied by enhancement of two negative peaks at 208 and 222 nm in the CD spectra, suggesting that the secondary structure of TP4 peptide contained an α-helix. Based on Figure 6E results, TP4 peptide dissolved in SDS solution (SDS 100 mM) at different temperatures (Figure 6G), NaCl concentrations (Figure 6F) or disodium EDTA concentrations (Figure 6H) all maintained a consistent secondary structure. The results showed that the TP4 peptide is stable and its secondary structure is maintained under many experimental conditions.

### Safety of the TP4 Microbicide Formulation in C57BL/6 Female Mice

**Figure 7:**
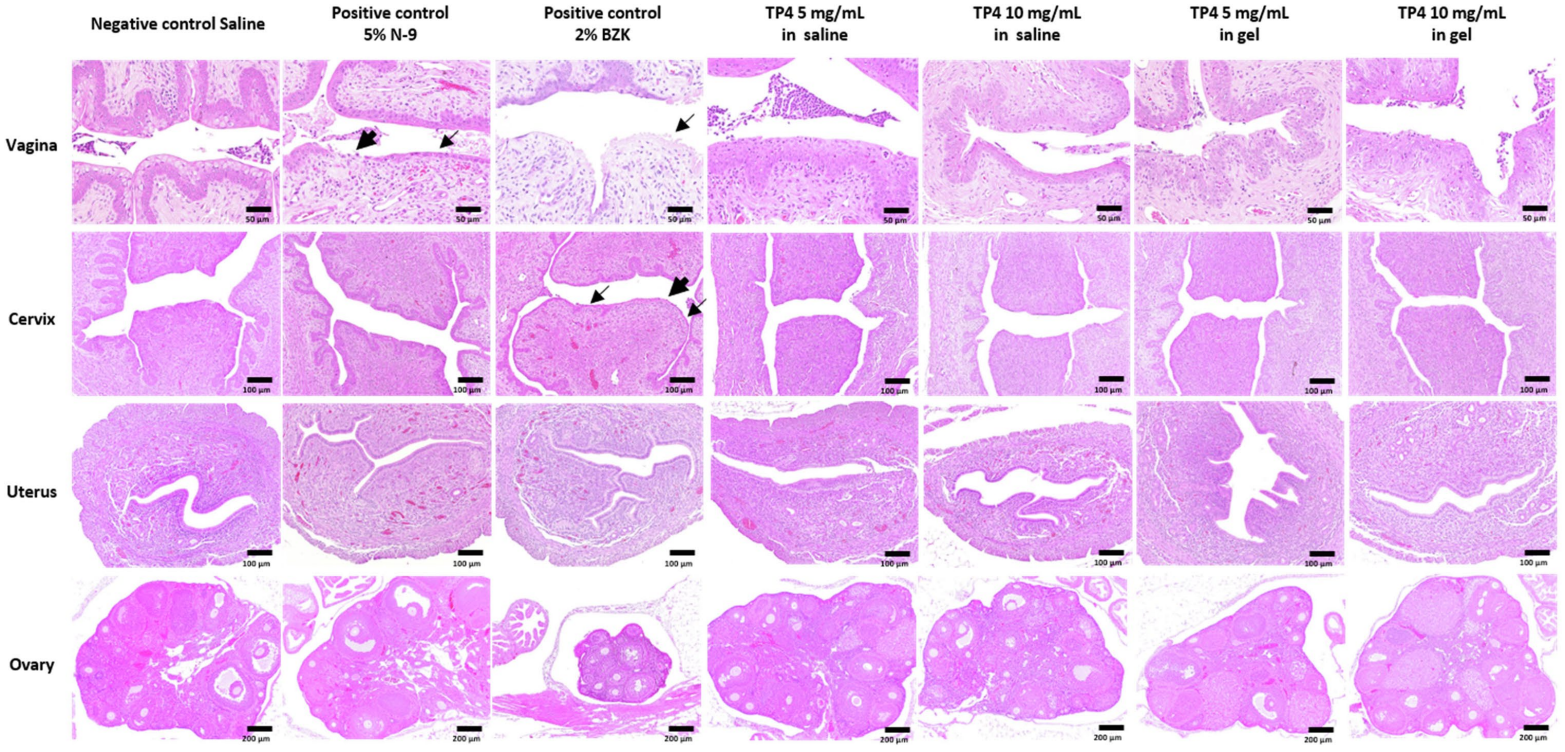
Tissue toxicity following vaginal administration of TP4 microbicide formulation in mice. Eight-week-old female C57BL/6 mice were used to evaluate tissue toxicity following vaginal administration of TP4. Hematoxylin and eosin (H&E) staining of mouse reproductive organs excised 24 h after intravaginal administration of saline (negative control), 5% N-9 (positive control), 2% BZK (positive control) and TP4 at 5 or 10 mg/ml in saline or gel (TP4 microbicide formulation). Vehicle gel contains 0.1% chitosan and 0.4 mM disodium EDTA in saline. Epithelial thinning and erosion are indicated by fine arrows and arrows, respectively. Images are representative of *n* = 5 mice. Scale bar applies to all images.

Following the evaluation of TP4 peptide combined with disodium EDTA and chitosan on BV-associated bacteria biofilms, the *in vivo* safety of the same TP4 microbicide formulation (TP4 peptide in 0.1% chitosan with 0.4 mM disodium EDTA in saline) was tested using C57BL/6 mice. The diestrus status of Depo-Provera-treated mice was assessed by vaginal cytology before drug exposure. As shown in Supplementary Figure 5A, leukocytes were predominantly present in vaginal fluid of mice 4 days after Depo-Provera administration, indicating a diestrus-like state. After synchronization, mice received a single vaginal administration of the test drug. Mice were sacrificed 24 h after drug administration for histopathological examination. N-9 is a non-ionic detergent microbicide that failed in Phase 3 trials because it causes damage to the vaginal epithelium and increases pro-inflammatory mediators that subsequently increase risk of HIV and gonorrhoea infection (Hoffman et al., 2004; Obiero et al., 2012). BZK is also known to damage epithelial tissues (Patton et al., 1999). The effects of N-9 and BZK on the genital tract are well-established (Ensign et al., 2012; Podaralla et al., 2014; Coulibaly et al., 2019), so we used these two agents as positive controls in our mouse vaginal irritation experiments. The histological analysis of reproductive organs is summarized in Figure 7 and Supplementary Table 2. No apparent lesions were observed in any reproductive organs of saline- or TP4-treated mice (including TP4 peptide and TP4 microbicide formulation). In contrast, vaginal irritation (epithelial thinning and erosion/necrosis) was observed in the 5% N-9 and 2% BZK groups (incidence in 5% N-9: 3/5; incidence in 2% BZK: 5/5). Furthermore, cervical irritation (epithelial thinning and erosion/necrosis) was observed in the 2% BZK group (incidence: 3/5). We also found that leukocytic exudate with cell debris, leukocyte infiltration of the subepithelial stroma, and mucification were present in both control mice and treated mice; these signs were considered to be related to the estrus cycle. The results suggest that intravaginal application of TP4 peptide (5 mg/ml and 10 mg/ml) or TP4 microbicide formulation (TP4 5 mg/ml and 10 mg/ml in gel) in mice once for 24 h did not cause toxicity or epithelial irritation in the female reproductive tissues (vagina, cervix, uterus, and ovary) when compared to known vaginal irritants (5% N-9 and 2% BZK).

### *In vivo* Effects of TP4 Microbicide Formulation

**Figure 8:**
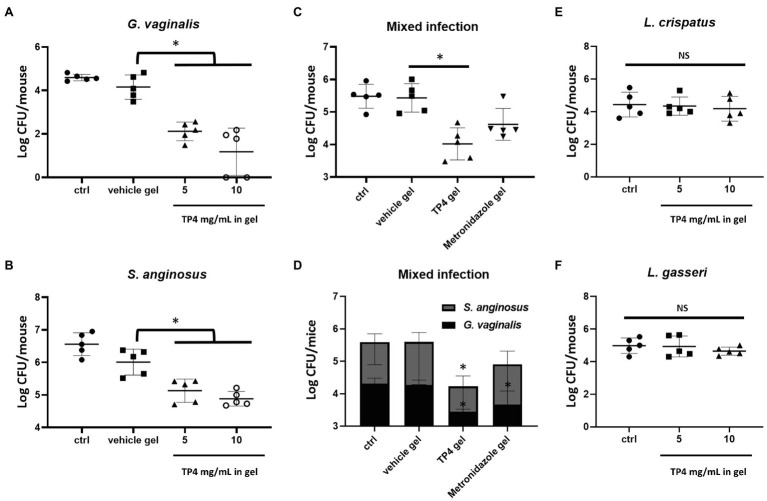
*In vivo* effects of TP4 microbicide formulation. Eight-week-old female C57BL/6 mice under pseudo-estrus conditions were vaginally treated two times per day with 20 μl vehicle gel or TP4 at 5 or 10 mg/ml in gel **(A,B)**, vehicle gel or TP4 or metronidazole (7.5 mg/ml) in gel **(C,D)**, and TP4 at 5 or 10 mg/ml in gel **(E,F)** after inoculation with *Gardnerella vaginalis* **(A)**, *Streptococcus anginosus* **(B)**, *Lactobacillus crispatus* **(E)**, *Lactobacillus gasseri* **(F)** or combined *G. vaginalis* and *S. anginosus* **(C,D)**. The results show the CFUs of *G. vaginalis* and *S. anginosus*. Vehicle gel contained 0.1% chitosan and 0.4 mM disodium EDTA in saline. Bacteria viability was assessed by CFU plate counts from vaginal lavage (150 μl vaginal lavage per mouse, *n* = 5 mice per group). All values represent the mean ± SD. ^*^p < 0.05 compared to vehicle gel **(A–D)** or ctrl **(E,F)**, determined by one-way ANOVA.

Finally, we investigated whether the TP4 microbicide formulation could be used to treat vaginal BV-associated bacteria challenges in mice. Treatment with β-estradiol improves the vaginal susceptibility of mice to infections and can be used in mouse models to evaluate the effectiveness of topical microbicides (Jerse et al., 2011; Gilbert et al., 2013). The synchronization of estrus status in β-estradiol-treated mice was assessed by vaginal cytology before treatments were administered. As shown in Supplementary Figure 5B, nucleated epithelial cells were predominant in vaginal fluid of mice 2 days after β-estradiol administration, indicating an estrus-like state. The mouse vaginal infection procedure and doses of bacterial strain inoculations are shown in Supplementary Figure 6. The β-estradiol-treated mice were challenged with *G. vaginalis* or *S. anginosus*. Of note, *S. anginosus* seemed to survive better in the mouse vagina compared to *G. vaginalis*. The inoculated doses of *G. vaginalis* and *S. anginosus* were respectively about 10^7^ and 10^6^ cells per day for each mouse (Supplementary Figure 6B). However, after 2 consecutive days of vaginal inoculations, the *G. vaginalis* and *S. anginosus* in the vagina of mice were respectively estimated to be about 4.1 × 10^4^ and 4.6 × 10^6^ cells per mouse (Figures 8A,B). Application of the TP4 microbicide formulation significantly decreased amount of *G. vaginalis* (Figure 8A) and *S. anginosus* (Figure 8B) recovered from mice vaginal lavage in a dose-dependent manner (TP4 0, 5, and 10 mg in vehicle gel).

In most cases of BV, at least two different pathogens are present in the vaginal epithelium (Castro et al., 2019). Therefore, we next investigated the effects of TP4 microbicide formulation in vaginas treated with mixed *G. vaginalis* and *S. anginosus*. TP4 was compared to metronidazole, which was prepared in the same vehicle gel. As shown in Figure 8C, the TP4 microbicide formulation significant decreased the total number of bacterial CFU recovered from mice vaginal lavage. In contrast, metronidazole-treated mice did not exhibit a significant difference with the vehicle group (Figure 8C), probably because it is inactivated on *S. anginosus* (Figure 8D). Next, we evaluated whether the TP4 microbicide formulation exhibited toxicity toward beneficial human vaginal lactobacilli. As shown in Figures 8E,F, no significant difference was found between TP4 microbicide formulation- and vehicle gel-treated mice that were inoculated with *L. crispatus* (Figure 8E) or *L. gasseri* (Figure 8F). Taken together, these results show that the TP4 microbicide formulation is effective at reducing BV-associated bacteria number and non-toxic to beneficial human vaginal lactobacilli *in vivo*.

## Discussion

In this study, we investigate the therapeutic potential of a broad-spectrum bactericidal peptide for treating BV-associated bacteria, especially in the form of biofilms. Our findings of a biofilm-disrupting TP4 microbicide formulation may provide a new strategy for future BV treatment. The current first-line antibiotics, metronidazole, and clindamycin, cannot inhibit many BV-associated bacteria (Schuyler et al., 2016; Table 1). In the past, BV was thought to be dominated by *G. vaginalis*, accompanied by other anaerobic bacteria, such as *Atopobium vaginae*, *Corynebacterium amycolatum*, *Prevotella bivia*, and *Fusobacterium nucleatum* (Spiegel et al., 1983; Deng et al., 2018). However, recent studies show that aerobic bacteria, such as *S. anginosus*, are also involved in vaginitis (Tao et al., 2019). This means that current first-line antibiotic regimens, which are only effective against anaerobic bacteria, are insufficient to treat some patients. However, the development of new BV medications has been extremely slow. Although, the FDA approved Secnidazole (Solosec, a nitroimidazole derivative) in 2017, this new drug is expected to suffer from the same drawbacks as current first-line antibiotics (Aziz et al., 2019).

The TP4 peptide has broad-spectrum antimicrobial and antibiofilm activity against relevant vaginal pathogens, making it different from metronidazole and clindamycin. In particular, the lactobacilli found in healthy human vaginas are resistant to TP4 peptide (Tables 1, 2; Figure 1). We speculate that the selective bactericidal activity of TP4 may be related to differences in cell membrane components, such as teichuronic acid, lipopolysaccharides, proteins, and phospholipids, that contribute to the overall surface charge and influence the electrostatic interactions between peptides and the membrane (Kłodzińska et al., 2010; Malmsten, 2016). *Gardnerella vaginalis* is one of the most important pathogens in BV (Schwebke et al., 2014), but its role has always been controversial because it is also present in healthy women (Hickey and Forney, 2014). Many reports show that compared with other BV-associated bacteria, *G. vaginalis* has a higher virulence potential, including cytotoxic effects and a strong propensity to form biofilms (Patterson et al., 2010; Machado et al., 2013; Alves et al., 2014). Our results demonstrated that *G. vaginalis* and *S. anginosus* exhibit some of the strongest biofilm-forming capacities among BV-associated bacteria (Table 1), which may promote biofilm structures that can promote growth of other BV-associated bacteria (Castro et al., 2019).

In BV, bacterial biofilms are formed by clusters of BV-associated bacteria that attach to the surface of vaginal epithelium cells (Castro et al., 2019) and are embedded in a self-produced matrix that includes proteins, polysaccharides, and extracellular DNA (Limoli et al., 2015). Studies have shown that biofilms persist on the vaginal epithelium even after putatively successful therapy with metronidazole (Swidsinski et al., 2008). The biofilms may provide a means for bacteria to evade host immune cells and reduce antimicrobial penetration. In this study, we found that the expression of drug resistance-associated genes, ABC transporter, and BcrA in the biofilm was significantly higher than in planktonic cultures for most clinical isolates (Supplementary Figure 3). This result is consistent with previous reports (Castro et al., 2017) and suggests that bacteria in biofilms may exhibit antibiotic resistance, which could explain the high recurrence rate with standard therapy. Therefore, new studies must focus on breaking down the biofilm structure to achieve optimal efficacy of antimicrobial therapies for BV. For instance, researchers have used DNase combined with metronidazole (Hymes et al., 2013) or amphoteric tenside pessary after metronidazole treatment to disrupt biofilms (Gottschick et al., 2017); however, this method still failed to prevent recurrence. Chitosan is an excellent excipient because it is cheap, non-toxic, and biodegradable. It has been widely used in biomedical and biotechnological fields (Muxika et al., 2017) due to its antifungal and biofilm-disrupting properties (Yang et al., 2017; Felipe et al., 2019; Araujo et al., 2021), which make it suitable as a vaginal drug carrier for BV treatment. In our study, we demonstrated that chitosan disrupted *G. vaginalis* biofilms, and TP4 peptide stored in chitosan for up to half a year still maintained its antibacterial activity without affecting the bactericidal activity of TP4 peptide on *G. vaginalis* (Figure 5). Therefore, chitosan may be highly useful as a vehicle or excipient for TP4 peptide to treat BV.

Antimicrobial peptides are rarely translated into clinical use due to their poor *in vivo* stability, quick degradation and inactivation by physiological environments. However, some candidate AMPs have entered clinical trials for use in treating local infections, such as LTX-109 (Lytixar; Clinical trial identifier: NCT01223222, NCT01803035, and NCT01158235), and Pexiganan (Locilex) (NCT01590758 and NCT01594762), SGX942 (NCT02013050; Mahlapuu et al., 2020). In order to evaluate the possible clinical use of TP4 peptides as a topical microbicide, one must first consider whether the local environment affects the bactericidal activity. Therefore, we performed a preformulation evaluation of the product (Alliance for Microbicide Development, 2006). Generally, AMPs are in a random coil conformation when dissolved in water and form an active amphipathic α-helix when they come into contact with the bacterial surface, subsequently promoting membrane leakage (Beevers and Dixon, 2010). The secondary structure is highly susceptible to environmental influences, which can change the ability of the AMP to cause membrane leakage (Júnior et al., 2018). We use VFS to mimic a vaginal fluid environment and found that in this solvent, TP4 maintains selective bactericidal activity for *G. vaginalis* and does not affect healthy human vaginal lactobacilli (Figure 1). We also considered peptide degradation during the manufacturing process or when it enters a biological system (Volkin et al., 2002). HPLC and CD were used to evaluate the TP4 peptide stability and secondary structure, revealing that TP4 peptide is stable over a wide range of temperatures, and concentrations of salt, disodium EDTA, hydrogen peroxide, and lactic acid; it does not lose its secondary structure under these conditions (Figure 6). Taken together, our data show that TP4 peptide has great potential for development as a microbicide candidate.

Various vaginal microbicide formulations are unable to enter clinical trials due to safety concerns, so lead microbicide formulations need to be evaluated early in the development process (Fernández-Romero et al., 2015). The WHO recommendations the osmolality of a microbicide formulation candidates should not exceed 380 mOsm/kg to reduce the risk of vaginal epithelium damage, and pH values should be about 3.5–4.5 (around normal vaginal values) to prevent increasing BV risk and HIV survival (World Health Organization, 2012). In this study, the formulation of chitosan as a TP4 peptide excipient conforms with these WHO recommendations (TP4 microbicide formulation osmolality <380 mOsm/Kg, pH 4.5). The preclinical safety of TP4 microbicide formulation was evaluated in C57BL/6 mice, including an assessment of vaginal inflammation, swelling, mucosa damage, and local toxicity, as well as harm to vagina normal flora (Fernández-Romero et al., 2015). There was no obvious toxicity in mice receiving intravaginal administration of the TP4 microbicide formulation. Estradiol-treated mice are often used as animal models to establish the colonization of pathogens associated with human reproductive tract infections, such as *G. vaginalis* (Gilbert et al., 2013; Hymes et al., 2013), *Streptococcus agalactiae* (Vrbanac et al., 2018), and *Neisseria gonorrhoeae* (Jerse et al., 2011). The pH of estradiol-treated mouse vagina (pH range 5.8–7.2; Jerse et al., 2011) is conducive to the colonization of BV-associated bacteria, which can be inoculated to evaluate the *in vivo* microbicidal activities of TP4 microbicide formulations. In this study, we established a murine model of single- or dual-species vaginal colonization by *G. vaginalis and S. anginosus*, or lactobacilli. We demonstrated that TP4 microbicide formulation decreases the colonization density of BV-associated bacteria but not lactobacilli. Not surprisingly, metronidazole did not reduce the *S. anginosus* survival in dual-species infected mice. However, the immunomodulatory effects of TP4 microbicide formulation could not be evaluated in this model because of a non-obvious inflammatory response in vaginal tissue and fluid (data not shown) in inoculated mice (observed after *G. vaginalis, S. anginosus*, or dual-species). These results are consistent with a previous report (Gilbert et al., 2013). Overall, this study suggests that the TP4 microbicide formulation has broad-spectrum BV-associated bactericidal activity. The TP4 peptide, chelating agent disodium EDTA and biofilm-disrupting chitosan might be a potent anti-biofilm formulation to treat polymicrobial BV biofilms.

## Data Availability Statement

The original contributions presented in the study are included in the article/**Supplementary Material**, further inquiries can be directed to the corresponding author.

## Ethics Statement

Mouse experiments were approved by the Academia Sinica Institutional Animal Care & Utilization Committee (Protocol number: IACUC 20-12-1568).

## Author Contributions

W-CL designed and performed the experiments, analyzed the data, and wrote the manuscript. Y-RC performed the Far-UV CD spectrum. C-MC designed the experimental studies. J-YC obtained funding, designed the experiments, and revised the paper. All authors contributed to the article and approved the submitted version.

## Funding

This work was supported by a grant through the National Biotechnology Research Park, Academia Sinica (NBRP-TRP-108-1-01). This research was supported by intramural funding from the Marine Research Station (Jiaushi, Ilan), Institute of Cellular and Organismic Biology, Academia Sinica to J-YC (Research Fellow).

## Conflict of Interest

The authors declare that the research was conducted in the absence of any commercial or financial relationships that could be construed as a potential conflict of interest.

## Publisher’s Note

All claims expressed in this article are solely those of the authors and do not necessarily represent those of their affiliated organizations, or those of the publisher, the editors and the reviewers. Any product that may be evaluated in this article, or claim that may be made by its manufacturer, is not guaranteed or endorsed by the publisher.
