# Supplementary material for: A Cationic Amphipathic Tilapia Piscidin 4 Peptide-Based Antimicrobial Formulation Promotes Eradication of Bacterial Vaginosis-Associated Bacterial Biofilms
Source: Front Microbiol. 2022 Mar 23;13:806654. doi: 10.3389/fmicb.2022.806654 (PMC9015711; doi:10.3389/fmicb.2022.806654)
Supplement: Supplementary file 1 [file Data_Sheet_1.docx]

**Supplementary information**

**Supplementary Figure 1. Secondary structure of cationic amphipathic TP4 peptide.** Illustration (upper) and electrostatic potential (lower) of the hydrophilic (A) and hydrophobic (B) faces of the amphipathic TP4 α-helix (Protein Data Bank accession number: 5H2S). The hydrophobic face contains hydrophobic residues distributed on one side of the helix (F, I, L, V, A, M; green). The hydrophilic face contains hydrophilic residues distributed on the other side of helix (H, K, R, S; blue). Surface electrostatic potential calculated by PyMOL. Positive, negative, and neutral electrostatic potentials are indicated by blue, red and white, respectively.

**Supplementary Figure 2. Trichomonacidal activity of TP4 peptide and metronidazole.** Growth of *trichomonads* in the presence of TP4 (A) and metronidazole (B). Initial inoculum cell numbers were 5 × 10^5^ cell/mL (dotted line). Viable cell counts of *trichomonas* after 24 hr incubation with different concentrations of TP4 (0-250 μg/mL) and metronidazole (0-31.25 μg/mL). All values represent the mean ± SD of three individual experiments. **p* < 0.05 compared to growth control (0 μg/mL).

**Supplementary Figure 3. Comparative transcription of virulence genes in *G. vaginalis* at biofilm and planktonic stage.** (A) Biofilm-forming capacities are shown as OD585, obtained after crystal violet staining. NS: No significant differences between *G. vaginalis* isolates. (B) Quantification of virulence gene transcription in *G. vaginalis* cultured under biofilm and planktonic conditions*.* The data represent relative genes expression in *G. vaginalis* biofilms cells compared to planktonic cells (dotted line). All values represent the mean ± SD of three individual experiments. **p* < 0.05 are significantly different between *G. vaginalis* cultured under biofilm and planktonic conditions were determined by one-way ANOVA.

**Supplementary Figure 4. Disodium EDTA influence on TP4 bactericidal activity in mature BV-associated bacterial biofilms.** Checkerboard analysis shows the biofilm cell viability and the combined effect of disodium EDTA and TP4 against BV-associated bacteria biofilms formed by *G. vaginalis* (A)*, S. anginosus* (B) and mixed cultures of both pathogens (C). Alamar Blue assay was used for quantitative biofilm cell viability. The heat plot shows the average of three replicates.

**Supplementary Figure 5. Vaginal cytology to identify estrous stage.**Vaginal smears were taken from 8-week-old C57BL/6 female mice four days after the administration of Depo-Provera (A) and mice two days after the administration of β-estradiol (B). The mice vaginal smears contained a prominent leukocyte population in (A), indicating a diestrus-like state. The mice vaginal smears exhibited predominant nucleated epithelial cells in (B), indicating an estrus-like state. Black arrows in (A) mark representative leukocytes. White arrows in (B) mark representative nucleated epithelial cells. Photomicrographs (100× magnification for all images; scale bar = 50 μm.).

**Supplementary Figure 6. *In vivo* evaluation of TP4 microbicide formulation efficacy.** (A) Schematic diagram of the experimental procedure for the mouse vaginal infection model. (B) List of bacterial strains, inocula amounts and test drug groups used in the mouse vaginal infection experiment. Vehicle gel contains 0.1% chitosan and 0.4 mM disodium EDTA in saline. S^R^: spontaneous streptomycin-resistant mutant.
