# Supplementary material for: A Cationic Amphipathic Tilapia Piscidin 4 Peptide-Based Antimicrobial Formulation Promotes Eradication of Bacterial Vaginosis-Associated Bacterial Biofilms
Source: Front Microbiol. 2022 Mar 23;13:806654. doi: 10.3389/fmicb.2022.806654 (PMC9015711; doi:10.3389/fmicb.2022.806654)
Supplement: Supplementary file 2 [file Table_1.docx]

**Supplementary Table 1.** List of primers used in this study.

| **Gene** | **Sequence (5’-3’)** | **Amplicon size (bp)** | **Reference or GenBank accession number** | **Experiments** |
| --- | --- | --- | --- | --- |
| *Gardnerella vaginalis* 16S | F: TGAGTAATGCGTGACCAACC | 167 | (Castro et al., 2019; Castro et al., 2017) | Used to quantify transcription of virulence genes in *G. vaginalis* |
|  | R: AGCCTAGGTGGGCCATTACC |  |  |  |
| Sialidase | F: CCGAATTTGCGATTTCTTCT | 189 |  |  |
|  | R: CGTACGGAAGTTTTGGAAGC |  |  |  |
| Multidrug resistance ABC transporter (ABC transporter) | F: CAGCACCTGTAGCTCCAACA | 195 |  |  |
|  | R: TGGCTCAAGAGATTGTGTGC |  |  |  |
| Bacitracin transport ATP-binding protein (BcrA) | F: CCGACCGCATACCTATTTTG | 178 |  |  |
|  | R: GCAAGACGGTCTCCAAACTC |  |  |  |
| Vaginolysin | F: GAACAGCTGGGCTAGAGGTG | 153 |  |  |
|  | R: AATTCCATCGCATTCTCCAG |  |  |  |
| Universal 16S | F: AGAGTTTGATCMTGGCTCAG | 309 | (Fukuda et al., 2016) | Used to identify inoculated mouse vaginal bacteria |
|  | R: ACTGCTGCSYCCCGTAGGAGTCT |  |  |  |
| *Lactobacillus gasseri* 16S | F: AAGGGCGCATGGTGAATGCCT | 312 | AF182721.1 |  |
|  | R: TGCTATCGCTTCAAGTGCTT |  |  |  |
| *Lactobacillus crispatus* 16S | F: GCGAGCGGAACTAACAGATTT | 150 | MT613437.1 |  |
|  | R: TGATCATGCGATCTGCTTTCT |  |  |  |
| *Streptococcus anginosus* 16S | F: GTTTTGCAGAAGCGATTGTC | 159 | AH008392.2 |  |
|  | R: TGAGAGCACGGTTTGAGTCG |  |  |  |
| *Gardnerella vaginalis* 16S | F: GGTCGCGTCCTATCAGCTTGTAG | 558 | MT644518.1 |  |
|  | R: GGACTACCAGGGTATCTAATCCT |  |  |  |
| F: forward, R: reverse | | | | |
