# Supplementary material for: A Cationic Amphipathic Tilapia Piscidin 4 Peptide-Based Antimicrobial Formulation Promotes Eradication of Bacterial Vaginosis-Associated Bacterial Biofilms
Source: Front Microbiol. 2022 Mar 23;13:806654. doi: 10.3389/fmicb.2022.806654 (PMC9015711; doi:10.3389/fmicb.2022.806654)
Supplement: Supplementary file 3 [file Table_2.doc]

**Supplementary Table 2.** Histopathology incidence table for reproductive organs after vaginal administration of TP4 microbicide formulation.
