# Supplementary figures and images for: A Cationic Amphipathic Tilapia Piscidin 4 Peptide-Based Antimicrobial Formulation Promotes Eradication of Bacterial Vaginosis-Associated Bacterial Biofilms
Source: Front Microbiol. 2022 Mar 23;13:806654. doi: 10.3389/fmicb.2022.806654 (PMC9015711; doi:10.3389/fmicb.2022.806654)

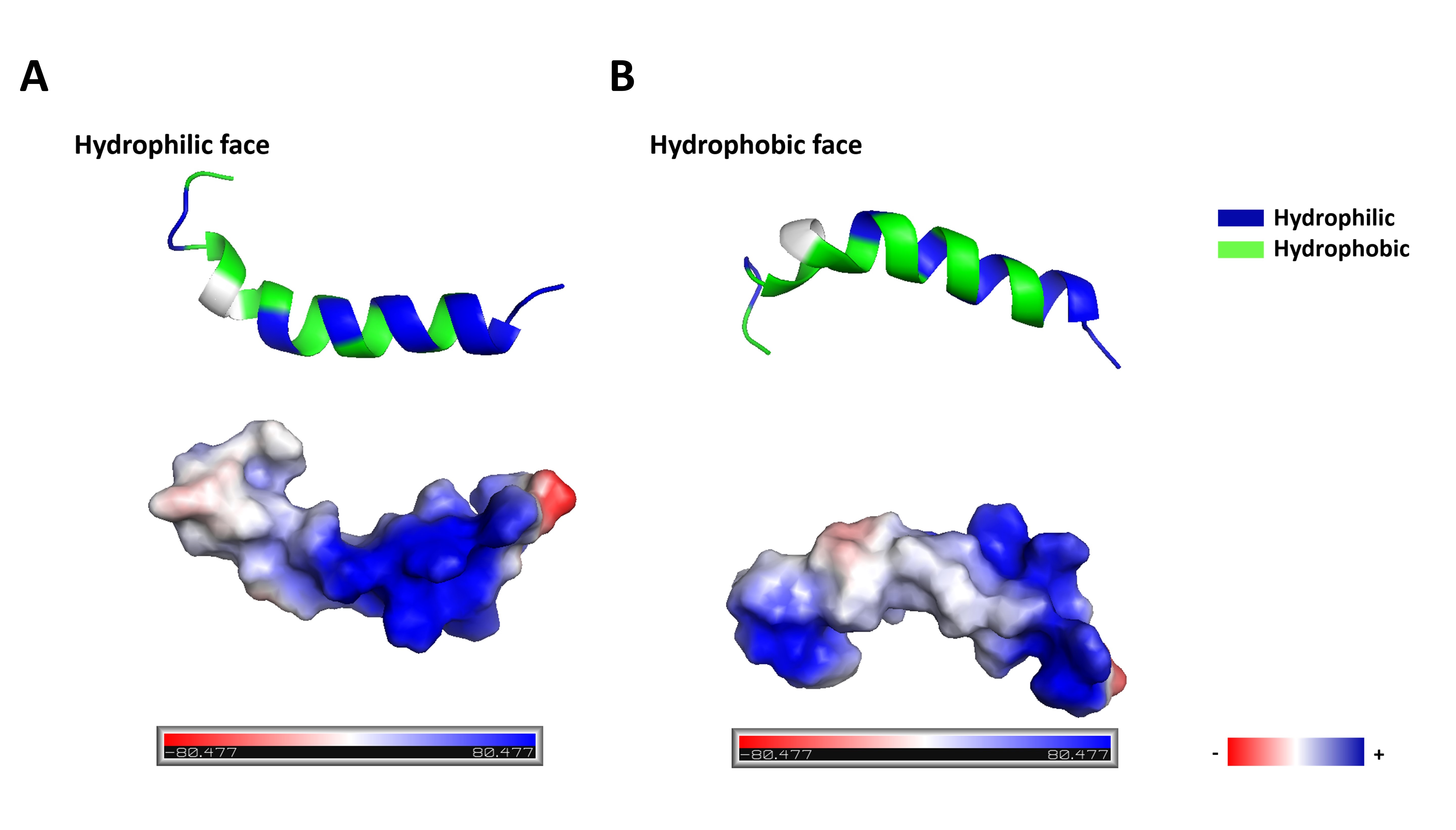

Supplement: Supplementary file 4 [file Image_1.JPEG]

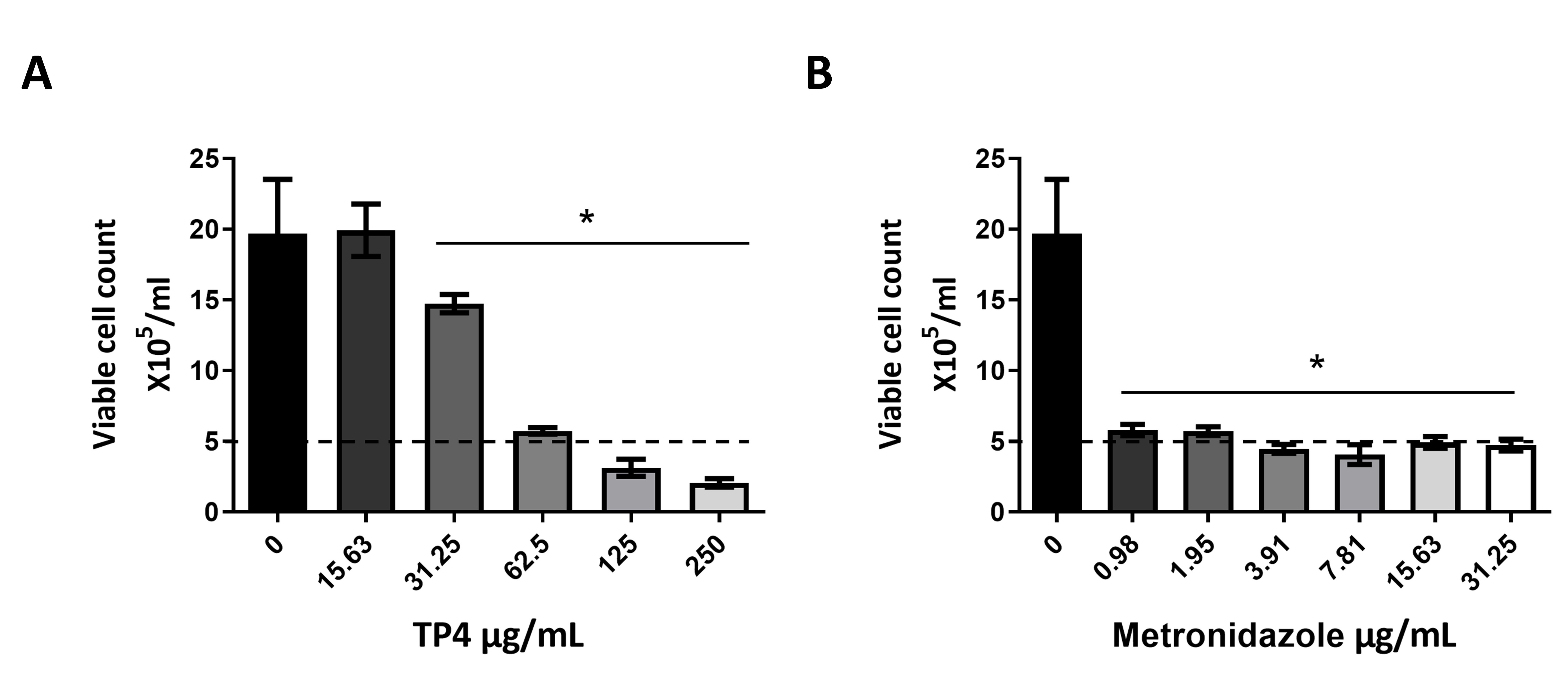

Supplement: Supplementary file 5 [file Image_2.JPEG]

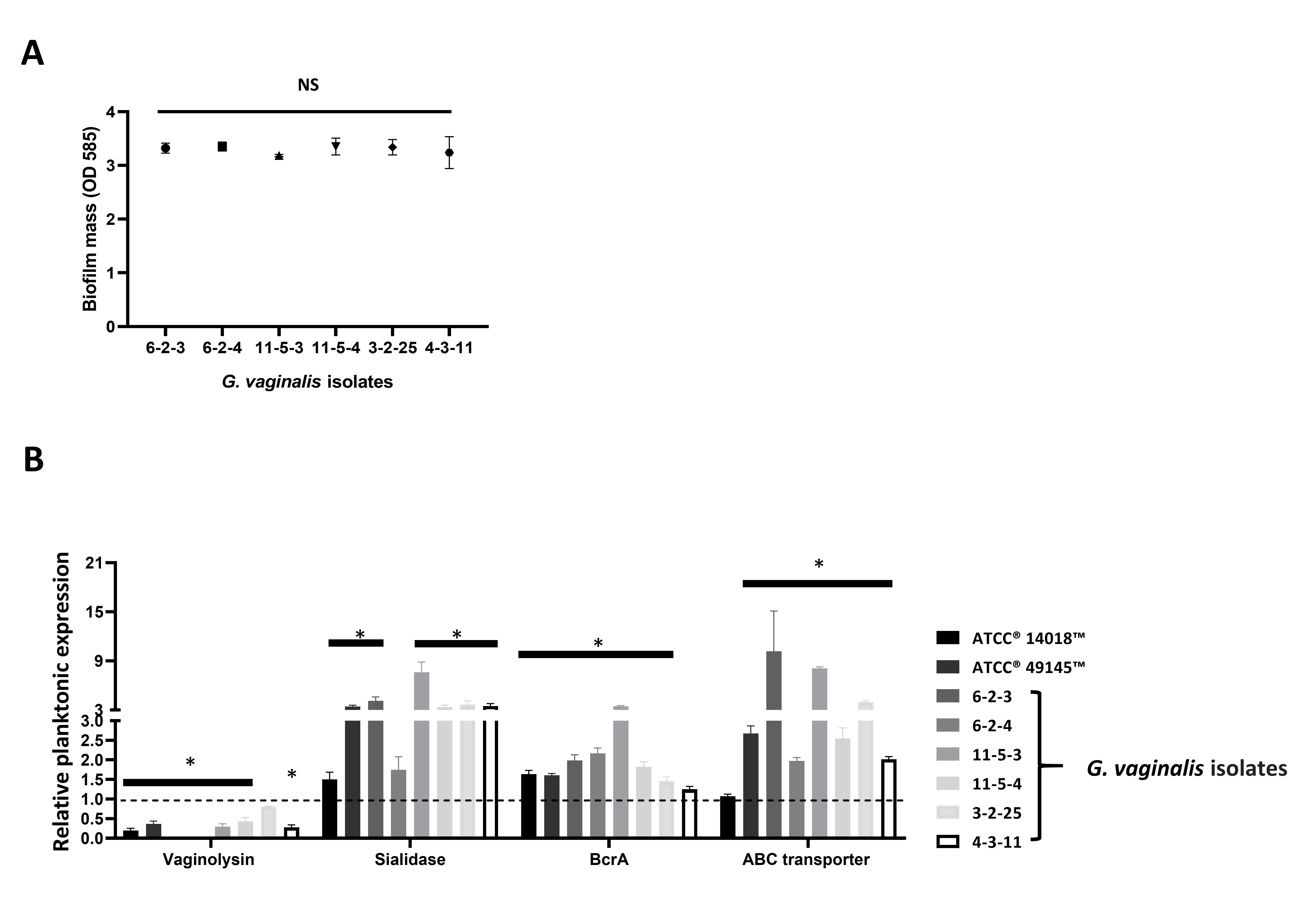

Supplement: Supplementary file 6 [file Image_3.JPEG]

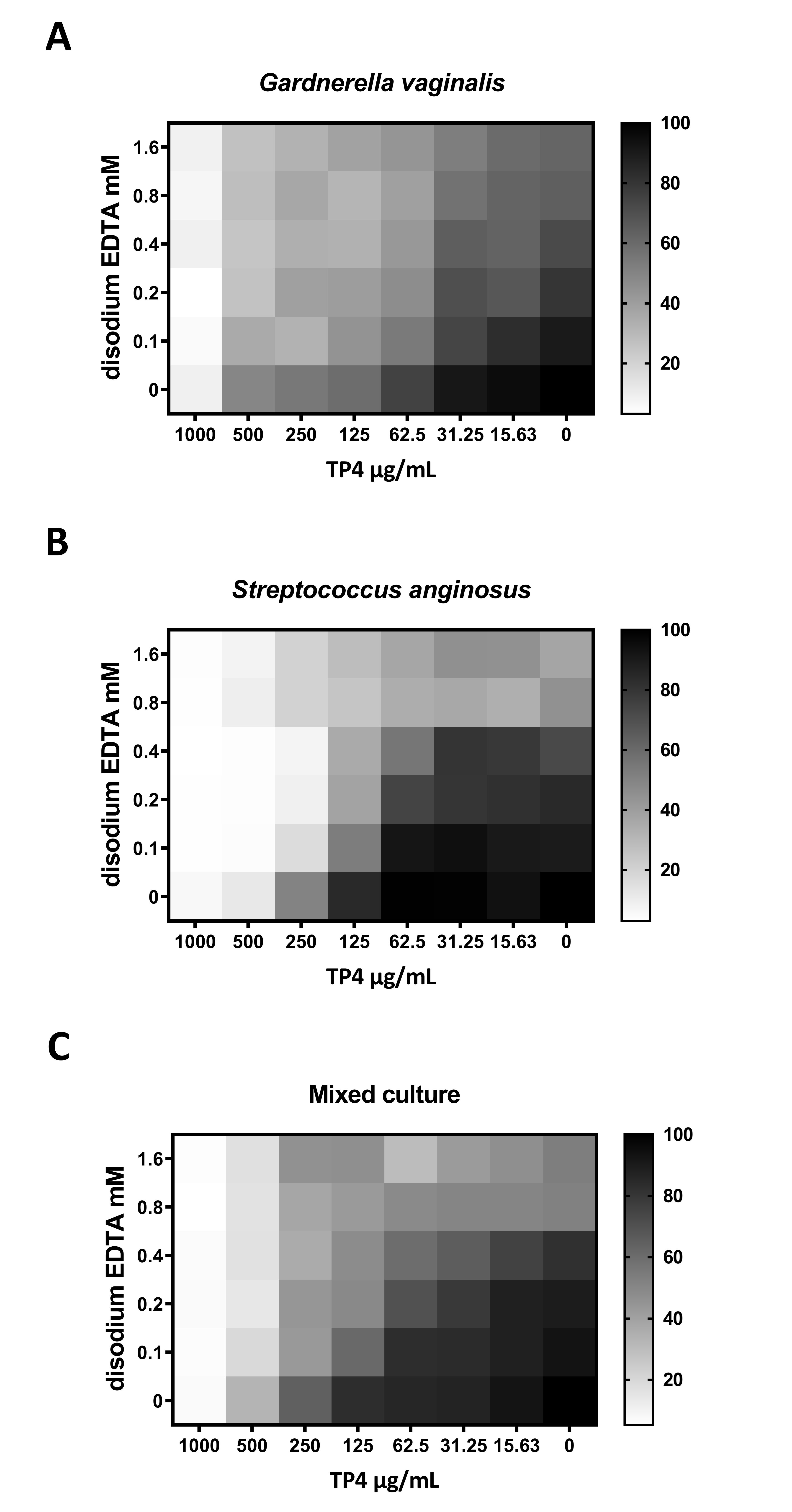

Supplement: Supplementary file 7 [file Image_4.JPEG]

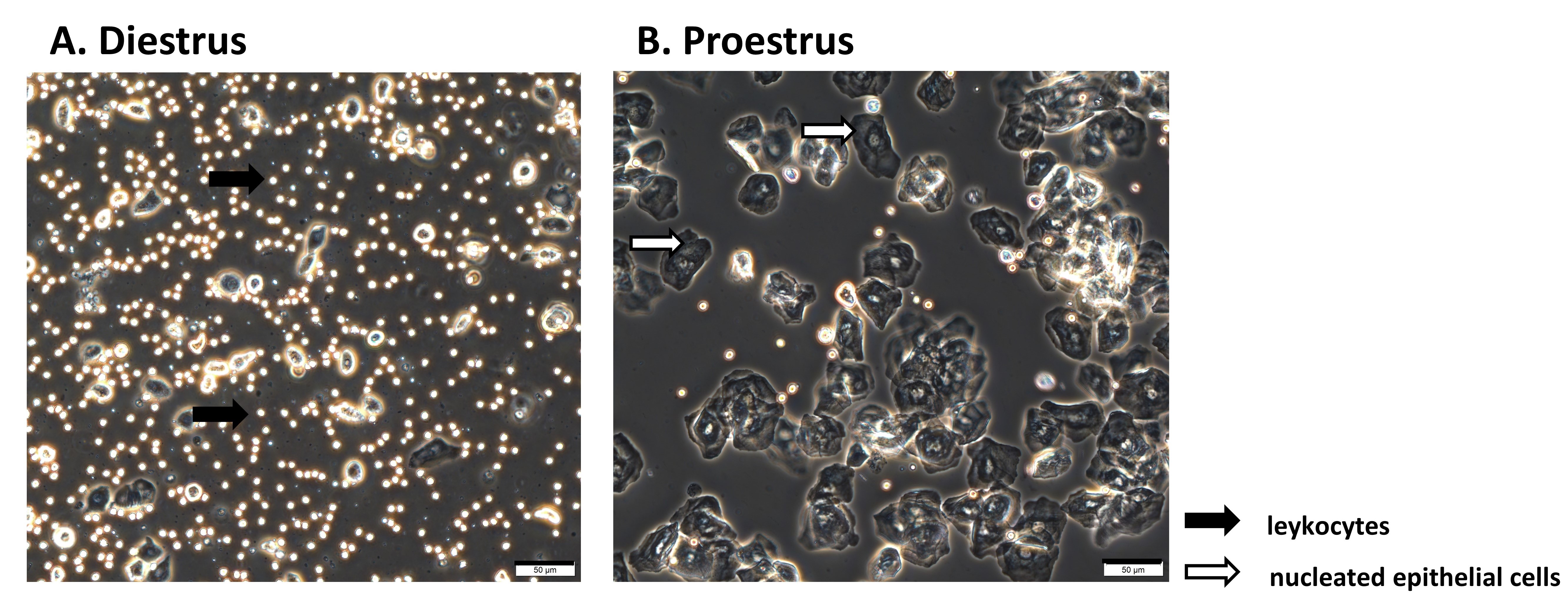

Supplement: Supplementary file 8 [file Image_5.JPEG]

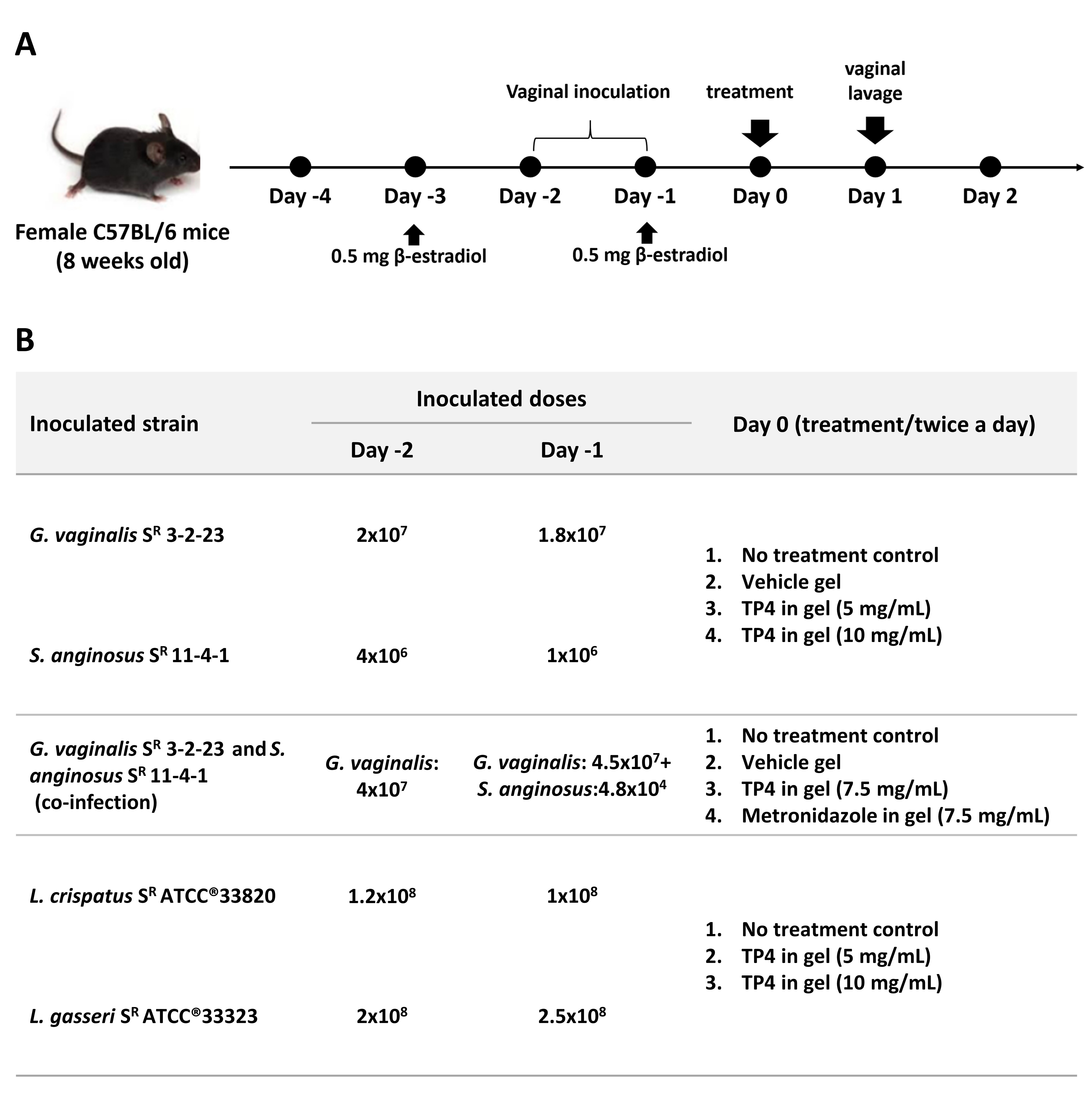

Supplement: Supplementary file 9 [file Image_6.JPEG]
